# Modified magnetic Nano-Biocomposite as friendly environmental catalyst for rapid degradation of organic dyes and selective aerobic oxidation of cyclohexene through advance oxidation process

**DOI:** 10.1016/j.heliyon.2024.e38453

**Published:** 2024-09-26

**Authors:** Maryam Lotfi, Alireza Faraji, Fatemeh Ashouri

**Affiliations:** a Active Pharmaceutical Ingredients Research Center, Tehran Medical Sciences, Islamic Azad University, Tehran, Iran; b Department of Organic Chemistry, Faculty of Pharmaceutical Chemistry, Tehran Medical Sciences, Islamic Azad University, Tehran, Iran; c Nutrition and Food Sciences Research Center, Tehran Medical Sciences, Islamic Azad University, Tehran, Iran; d Department of Applied Chemistry, Faculty of Pharmaceutical Chemistry, Tehran Medical Sciences, Islamic Azad University, Tehran, Iran

**Keywords:** Nano-biocomposite, Day degradation, Aerobic oxidation, Allylic oxidation

## Abstract

To eliminate contaminated organic matter from water and wastewater, a stable, recyclable, and environmentally friendly nano-biocomposite was designed. The magnetic Fe_3_O_4_ nanoparticles were functionalized by SiO_2_/N-*2*-(aminoethyl)-3-aminopropyl/glutaraldehyde/chitosan/Cobalt to fabricate nano-biocomposite (FS-(Am/*g*/Cs)@CoNPs). The morphological/structural identification of nano-biocomposite was carried out by ICP-OES, DR-UV, XRD, FE-SEM, TEM, HR-TEM, BET, EDX, FT-IR, TGA, and VSM techniques. The catalytic performance of this heterogeneous catalyst was studied in the degradation of organic dyes including methylene blue, methyl orange, methylene violet, and Bisphenol A, and efficiency was achieved at 99.3 % (*k*_obs_ = 0.3703 min^−1^), 97.4 % (*k*_obs_ = 0.3627 min^−1^), 93.6 % (*k*_obs_ = 0.228 min^−1^) and 98.8 % (*k*_obs_ = 0.459 min^−1^) after 12–14 min at room temperature in pH = 7.0, respectively. The reaction proceeded through the activation of Co^2+^/Co^3+^ which occurs on the surface of the FS-(Am/g/Cs)@CoNP by the PMS/O_2_ system through recombination of SO_4_^•-^ and OH^•^. Furthermore, selective allylic oxidations of cyclohexene to cyclohexenone (TOF = 4583.7 h^−1^, 100 % selectivity, 98 % conversion) were done by this nano-biocomposite.

## Highlights

• The Nano-biocomposite was synthesized by functionalization of magnetic-silica@(N-*2*-(aminoethyl)-3-aminopropyl) support with a chitosan/glutaraldehyde to facilitate cobalt immobilization.
• The nano-biocomposite was exhibited excellent efficiency, stability, and reusability in rapid decolorization of organic dyes.
• The selective production of cyclohexenone was done using designed nano-biocomposite in mild reaction conditions in the absence of any reducing agents and promotors.
• The short response time at room temperature, suggesting the potential of applications of nano-biocomposite in green environmental management.

## Graphical abstract

**Figure:**
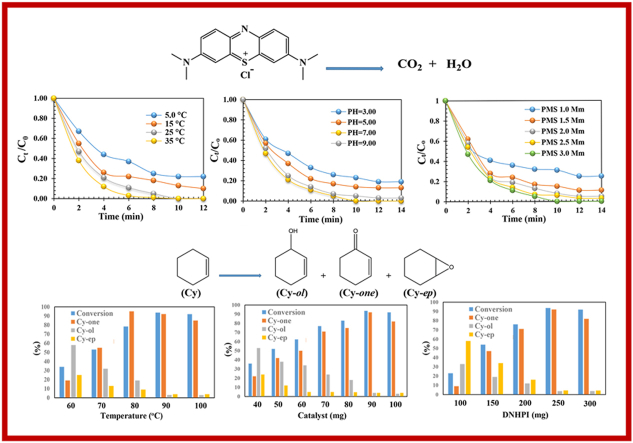

## 1 Introduction

The increase in the population and the development of various chemical industries have dramatic impacts on the environment and ecosystem. The industries including textile, chemical, pharmaceutical, paper, dyeing, mining, printing, paint, veterinary medicine, and food industries, discharge an extensive range of toxic municipal and industrial waste causing a serious environmental threat [1]. Among industrial chemicals that make water unhealthy, dyes including methylene blue (MeB), Methyl Orange (MO), Methylene Violet (MV), and Bisphenol A (Bis A) are widely applied [[2], [3], [4], [5]]. Almost most of these dyes are non-biodegradable, toxic, carcinogenic, persistent, and pose a serious threat to human health and aquatic ecology [1,2,6]. Therefore, the degradation of non-biodegradable, toxic pollutants and waste to harmless and value-added matters is a major concern of environmentalists [2,7,8].

Hence, dye-containing wastewater should be treated effectively using techniques such as adsorption [9], biosorption [10], coagulation [11,12] electrocoagulation [13], vacuum membrane distillation [14], phytoremediation [15,16], liquid-liquid extraction [17], ultrafiltration [18], nanofiltration [19], microwave treatment [20], biodegradation [21], ozonation membrane [22], photolysis with UV & H_2_O_2_ [9,23], electrochemical [24], photo-Fenton [25], photocatalytic removal [9,26] and catalytic oxidation [27] to prevent the mentioned impacts. Each of these methods has its unique advantages, but the advanced oxidation processes (AOPs) have been highly considered due to their high efficiency and non-production of toxic by-products [4,9,28].

On the other hand, Cyclohexenone is an extensively used building block in organic synthesis chemistry, as it offers many diverse ways to extend molecular frameworks. Therefore, allylic oxidation of cyclohexene is a favorable method for the production of *α,β*-unsaturated cyclohexenone as an intermediate for the making of surfactants, polymers, agrochemicals, and drugs but this process encountered difficulties in selectivity control, recovery, and environmentally friendly [[29], [30], [31]]. Thus, to achieve environment-conscious chemical processes many attempts are to develop heterogeneous systems that can recover facilely and reuse effectively.

In this respect, chitosan and modified chitosan are some of the most potential renewable green materials as bio-catalysts due to (*i*) their abundant availability and biodegradable ability; (*ii*) their flexible structure and hydroxyl, primary amino, and acetylamino functional groups as absorbents for metals; (*iii*) their good thermal and chemical stability; (*iv*) easy functionalizations through amine group, and)*v*) hydrophilic and basic moieties [32]. Thus, it could be possible to apply the chitosan to achieve the bio-composite and use it as a precursor to developing a bimetallic catalyst for the activation of PMS/O_2_.

Here, we fabricated the magnetic core-shell beads with amino groups, glutaraldehyde, and chitosan to provide a FS-(Am/*g*/Cs) as an eco-friendly activator with excellent chemical and mechanical strength that can be impregnated with Co(II) NPs and provide a sustainable activator an FS-(Am/*g*/Cs)@CoNP- to activate PMS and O_2_ for the degradation of organic dyes and selective aerobic oxidation of cyclohexene, respectively. The Nano-biocomposite structure was well-defined and, the effect of critical parameters, and coexisting ions on the process was systemically examined.

## 2 Experimental Section

### 2.1 Materials and instruments

Iron (II)-chloride tetrahydrate (FeCl_2_ · 4H_2_O), Iron (III)-chloride hexahydrate (FeCl_3_ · 6H_2_O), Hydrochloric acid (HCl, 37 %), Sodium hydroxide solution (NaOH, 1.5 M), Ethanol (EtOH), Ammonia solution (NH_3,_ 25 %), Tetraethyl orthosilicate (TEOS, Si(OC_2_H_5_)_4_), N-(2-Aminoethyl)-3-aminopropyltrimethoxysilan (AEPMS, H_2_N(CH_2_)_2_NH(CH_2_)_3_Si(OCH_3_)_3_), Acetic acid (CH_3_CO_2_H), Chitosan, Glutaraldehyde solution (OHC(CH_2_)_3_CHO, 25 %) and Cobalt (II) acetate (II) (CH_3_CO_2_)_2_Co) were obtained from Merck, Fluka and Sigma-Aldrich Co., Ltd., and utilized without further purification. Potassium peroxymonosulfate (PMS, KHSO_5_ · 0.5KHSO_4_ · 0.5K_2_SO_4_), methylene blue (MeB, C_16_H_18_ClN_3_S_3_H_2_O), N, N-dihydroxypyromellitimide (DNHPI, C_10_H_6_N_2_O_6_) and Cyclohexene (CY, C_6_H_10_) were purchased from Sigma-Aldrich.

Also, the instrument for the characterization and analysis of products are mentioned below: ICP-OES (SPECTRO ARCOS, Germany); EDS (MIRA II, with France SAMX detector the Czech Republic); Brunauer–Emmett–Teller (BET; BELSORP MINI II, BEL, Japan); TEM (Philips 501 microscope,80 kV voltage); SEM (Tecnai F30TEM operating at 300 Kv); DLS(SZ100, Horiba, Japan); FT-IR (Shimadzu Varian 4300 Fourier Transform Infrared spectrometer, KBr pellets); UV-DRS (UV- 160 A, Shimadzu, Japan) TGA (PerkinElmer TG-DTA 6300, heating rate of 15 °C/min); XRD (Bruker D8 Advance diffractometer, CuKα radiation, 40 Kv, 20 mA) & VSM (BHV-55 VSM). GC & GC-MS (HP 6890/5973 GC/MS, Shimadzu GC-16A gas chromatograph (GL-16, 5 m −3 mm OV-17 column, 60–220 °C (10 °C/min), Inj. 230 °C, Det. 240 °C.

### 2.2 Preparation of FS-(Am/g/Cs)@Co nano-biocomposite

The typical synthesis procedures of magnetic Fe_3_O_4_ and Fe_3_O_4_@SiO_2_ nanoparticles were done via co-precipitation and Stöber sol-gel methods, respectively [31]. The solution included 25 mL EtOH/H_2_O (1.6 v/v) and 1.0 g Fe_3_O_4_ NPs were dispersed in 4.0 mL NH_3_ (25 *wt*%) solution. After sonicating for 15 min, the solution was vigorously stirred with 0.6 mL TEOS (Tetraethyl orthosilicate) at ambient temperature for 2 h. In the next step, a micro syringe pipette was applied to inject the AEPAS (N-(2-Aminoethyl)-3-aminopropyltrimethoxysilan) (0.1 mL) into the obtained solution, which was then mixed for the grafting of the N-et-NH_2_ group on the magnetic beads under a continuous mechanical string for 5.0 h. The modified magnetic bead, which is coded FS-Am NPs, was isolated using an external magnet, washed with H_2_O/EtOH (1:1 v/v) four times, and dried at 60 °C under a vacuum.

**Scheme 1:**
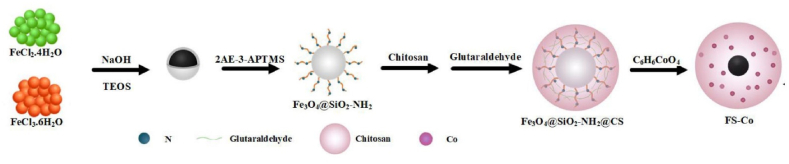
The schematic diagram of the synthesis process of FS-(Am/*g*/Cs)@CoNP Nano-biocomposite.

Afterward, FS-Am NPs (1.0 g) were dispersed in distilled water (50 mL) and added dropwise to a 30 mL chitosan/AcOH solution (0.001 w/v). Then, a 20 mL glutaraldehyde aqueous solution (5 wt%) as a cross-linking agent was added to the solution. After 4 h, the solid was isolated, washed with EtOH/H_2_O (5.0 mL, 1:1 v/v), and then dried in an oven at 60 °C. The obtained black solid (0.5 g) was mixed with 100 mL distilled H_2_O for 20 min by ultrasonic dispersion method. Afterward, the prepared solution was added to an aqueous solution of Co(C_2_H_3_O_2_)_2_ (25 × 10^−4^ w/v) and stirred for 24 h under reflux. Finally, the deep brown powder was extracted by an external magnet and washed with H_2_O/EtOH/acetone (2 × 5) and then dried in a vacuum oven at 60 °C for 4.0 h. The schematic diagram of the synthesis process of nano-biocomposite is shown in Scheme 1.

### 2.3 Catalytic efficacy of FS-(Am/g/Cs)@Co nano-biocomposite

#### 2.3.1 Catalytic degradation of dyes

The methylene blue dye as a target pollutant was used to evaluate the catalytic activity of FS- (Am/*g*/Cs)@CoNP. At first, by the Response Surface Methodology (RSM) program, 54 experiments were designed (S1, Table S4). The subsequent degradation studies were carried out in a batch condition. Various MeB dosages (5.0–25 mg/L) were added into several solutions with initial pH (3.0–9.0) and different amounts of FS-(Am/*g*/Cs)@CoNP (0.5–3.0 g/L) with PMS dosage (0.5–3.0 mM) at different temperatures (5–35 °C). The obtained solution was injected via a filter into a UV–vis spectrophotometer's Cuvette to measure MeB concentration. Eq. (1) was used to calculate the removal efficiency (RE, %):

(Eq.1) $$RE\;\left(\%\right)=\frac{C_{0}-C_{t}}{C_{0}}\times 100$$

C_0_- initial concentration of MeB.

C_t_ - the instantaneous concentration of MeB in the solution.

#### 2.3.2 Aerobic oxidation of cyclohexene

The FS-(Am/*g*/Cs)@CoNP (90.0 mg), N,N-dihydroxypyromellitimide (DNHPI) (250 mg), and cyclohexene (9.0 mmol) were added to the round bottom flask under O_2_. The mixture was stirred for 10 h at the optimal temperature. Then, the catalyst was separated by an external magnet, and the filtrate was analyzed by GC and GC–MS for identification and quantification of the products. The separated catalyst was washed with H_2_O/EtOH/ether (1:1:1), dried, and used for another similar reaction. The conversion (α) was calculated based on changes in the relative areas (%) of the CY and product peaks according to Eq. (2):

(Eq.2) $$\alpha .\;\%=\frac{\sum S,{\%}_{\text{products}}}{S,{\%}_{\text{CY}}+\sum S,{\%}_{\text{products}}}\times 100$$

The products are including cyclohexene-1-ol (Cy-*ol)*, cyclohexene-epoxide (Cy-*epo),* and cyclohexene-1-one (Cy-*one*). For each aerobic oxidation process, the selectivity (S) to ACP, was calculated according to Eq. (3):

(Eq.3) $$S_{CY-one,}\%=\frac{{S,\%\;}_{\text{CY}=O}}{{S,\%}_{\text{CY}=O+}\;{S,\%}_{\text{Other}\;\text{product}}}\times 100$$

As a significant parameter for the evaluation of dendritic catalyst efficiency, the turnover frequency (TOF) was calculated according to the below equations (Eq. (4)):

(Eq.4) $$\text{TOF}\;\left(h^{-1}\right)=\frac{\text{Mole}\;\text{of}\;\text{desired}\;\text{product}}{\text{Moles}\;\text{of}\;\text{Co}\;\text{in}\;\text{bio}-\text{composite}\times \text{reaction}\;\text{time}\;}$$

## 3 Result and discussion

### 3.1 Characterization of nano-biocomposite

**Fig. 1:**
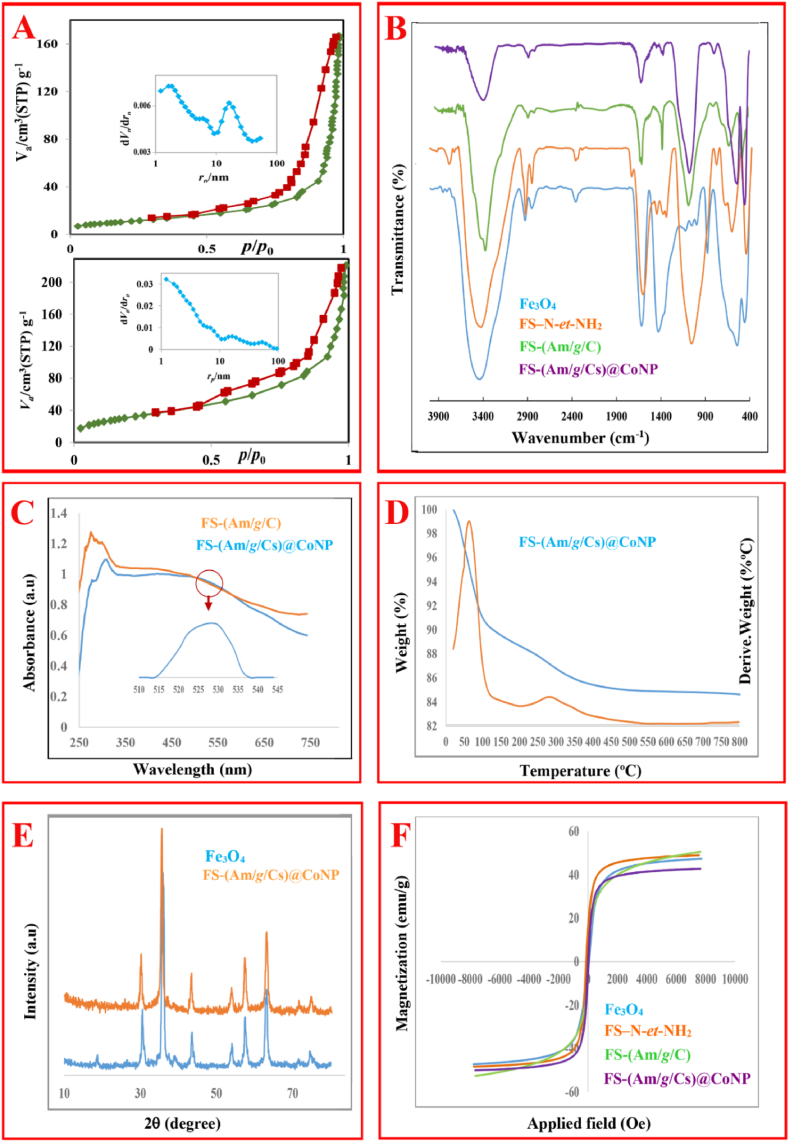
(**A**) BET analysis, (**B**) FT-IR, (**C**) Uv-DRS spectra, (**D**) TGA profile, (**E**) XRD pattern, (**F**) VSM of FSNPs, FS-(Am/*g*/Cs), & FS-(Am/*g*/Cs)@CoNP.

The structural parameters of the nano-biocomposite and its parents were also evaluated by N_2_ adsorption-desorption analysis (pore size, specific surface area, and porosity). As shown in Fig. 1A, the N_2_ adsorption-desorption isotherms of the magnetic core-shell and FS-(Am/*g*/Cs)@CoNP exhibited a typical type IV curve with an H4-type hysteresis loop [32]. Table 1 shows the S_*BET*_ (specific surface area BET), V_*BJH*_ (total pore volume), and *MPD* (mean pore diameter) of the magnetic bead and FS-(Am/*g*/Cs)@CoNP. The dominant interaction between adsorbate and the adsorbed layer causes to S_BET_ of FS-(Am/*g*/Cs)@CoNeP (115.5 m^2^ g^−1^) has be three-fold higher than a parent (38.8 m^2^ g^−1^).

**Table 1:** Morphology and chemical composition of FS-(Am/*g*/Cs)@CoNP Nano-biocomposite.

Samples	Elemental analysis (wt%)^a^		Co (%)^b^	Structural Parameters^c^		
	C	N		S_*BET*_ (m^2^/g)	V_*BJH*_ (cm^2^/g)	*MPD* (nm)
Fe_3_O_4_ NPs	–	–	–	38.82	0.174	6.67
Fe_3_O_4_@SiO_2_ NPs	–	–	–	53.51	0.149	13.2
FS-(Am/*g*/Cs)@CoNP(fresh)	29.2	5.30	1.4	65.5	0.933	27.3
FS-(Am/*g*/Cs)@CoNP^d^	30.1	5.34	1.35	69.3	0.942	29.4
FS-(Am/*g*/Cs)@CoNP^e^	39.2	6.23	1.02	75.5	0.956	37.1

a Carbon and Nitrogen were estimated from the elemental analyses.

b Cobalt content by ICP-OES.

c Pore size calculated using *BJH* method.

d Spent catalyst after 5 runs.

e Spent catalyst after 9 runs.

For identifying the functional group in nano-biocomposite, the FT-IR spectra of Fe_3_O_4_, FS–N-*et*-NH_2_, FS-(Am/*g*/C), and FS-(Am/*g*/Cs)@CoNP were evaluated (Fig. 1B). The typical absorption peaks were observed at 542.54 cm^−^^1^ for stretching vibrations of Fe- O- Fe, 462.93 cm^−^^1^ for O-Si-O bending, 792.12 cm^−^^1^ for Si-O-Si symmetric, and 1072.95 cm^−^^1^ for Si-O-Fe stretching vibrations. The overlapping of -NH and -OH stretching vibrations is attributed to a strong broadband at 3424.91 cm^−1^ [33]. The coating of magnetic chitosan is determined by the formation of peaks at 2924.01 and 1619.21 cm^−1^, which are related to the –CH– and N–H stretching vibrations of chitosan in FS-(Am/*g*/Cs)@CoNP, respectively [33]. Additionally, the peaks that appeared at 1384.22 and 1083.28 cm^−1^ were attributed to the C-N (amin) and C–O (ether) vibrational aromatic rings, respectively. The stretching vibration of the Co-O band is represented at 803.38 cm^−1^.

The induced electron delocalization was thermally happening in FS NPs between Fe ^2+^ and Fe ^3+^ ions. The peaks related to the Fe_3_O_4_-SiO_2_ core-shell appear at 800–2500 nm in FT-IR and 407 nm in the UV–Vis region (Fig. 1C) [34]. Surface plasmon resonances (SPRs) dominated the optical spectra of the metal nanoparticles, moving to longer wavelengths with an increase in particle size. Therefore, a broad peak at 520–530 nm (*d→d*∗) in the UV–Vis spectrum of FS-(Am/*g*/Cs)@CoNP (Fig. 1CA) was created due to stimulation in the surface plasmon vibrations of Cobalt nanoparticles [35].

Thermogravimetric analysis (TG-DTG) has often been employed to determine the functional group content and thermal durability. The weight loss of FS-(Am/*g*/Cs)@CoNP is about 15.37 % (1.031 mg) during the temperature range of 20–800 °C (Fig. 1D). In the first stage (<63.12 °C), the FS-(Am/*g*/Cs)@CoNP loosens the absorbed physical and/or chemical water. The weight loss was caused by the thermal decay of chitosan when the temperature was elevated above 262.50 °C. From 262.50 °C to 800 °C, there was no significant weight change, implying that it is durable up to 262.5 °C and the chemical structure of FS-(Am/*g*/Cs)@CoNP was well-retained within the temperature range [36].

The phase behavior and crystallinity of the FS-(Am/*g*/Cs)@CoNP were evaluated by XRD analysis. As shown in Fig. 1E, the diffraction reflexes of 35.73° (3 1 1), 62.86° (4 4 0), 30.42° (2 2 0), 57.56° (5 1 1), 43.43° (4 0 0), 53.58° (4 2 2) and 18.41° (1 1 1)**,** which correspond to the crystalline cubic spinel structure of FSNPs (JCPDS 65–3107) [6]. The reflexes of shells, silica (15°–30°), and chitosan (9°–10° and 20°–21°) illustrated that core-shell structure was successfully constructed. No impurity peaks were observed in the XRD pattern of FS-(Am/*g*/Cs)@CoNP.

The magnetic properties of synthesized samples were further evaluated by a magnetic hysteresis loop to master the response capacity of these nanoparticles to external magnets (Fig. 1F). The *Ms* (saturation magnetization) value of bare FSNPs is noted to be 48.08 emu/g, whereas the Ms of FS-Am, FS-Am/*g*/C and FS-(Am/*g*/Cs)@CoNP were determined to be 40.61 emu/g, 38.86 emu/g, and 25.89 emu/g. The significant decrease in *Ms* from FSNPs to FS-(Am/*g*/Cs)@CoNP is due, principally, to an increase in the proportion of inert shells (silica and chitosan) in the samples. Thereby, it can be concluded that FS-(Am/*g*/Cs)@CoNP has an excellent saturation magnetization due to its structure.

**Fig. 2:**
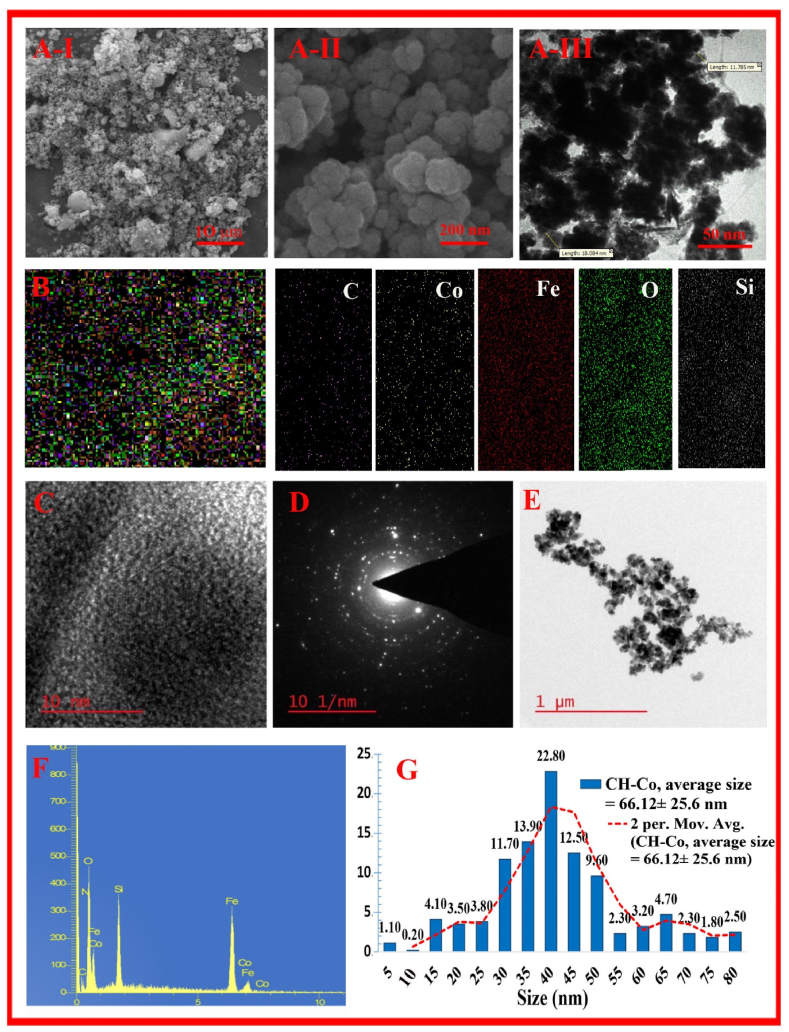
Microscopic analyses of SEM (**A**), SEM-map (**B**), TEM (**C**), and HR-TEM (**D**) images of fresh FS-(Am/*g*/Cs)@CoNP catalyst and TEM image (**E**) and EDS (**F**) and DLS analysis (**G**) of the recovered FS-(Am/*g*/Cs)@CoNP catalyst after 6 runs.

The TEM and SEM images of the FS-(Am/*g*/Cs)@CoNP confirmed that the synthesized Nano-biocomposite is in the nanometric range (40–50 nm) with small aggregation and a rough and porous morphology, which is consistent with the obtained results from DLS (Fig. 2). Moreover, the presence of C, N, Fe, Co, Si and O elements was studied by the EDS spectrum and FE-SEM images of FS-(Am/*g*/Cs)@CoNP (Fig. 2F).

### 3.2 Catalytic performance of nano-biocomposite

#### 3.2.1 Degradation of methylene blue dye

**Fig. 3:**
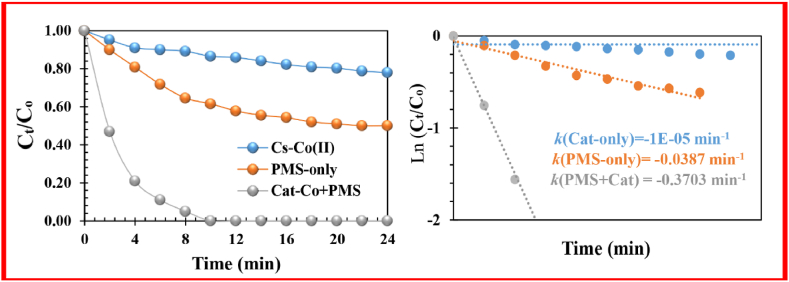
Degradation of MeB in the presence of different catalysts.

**Fig. 4:**
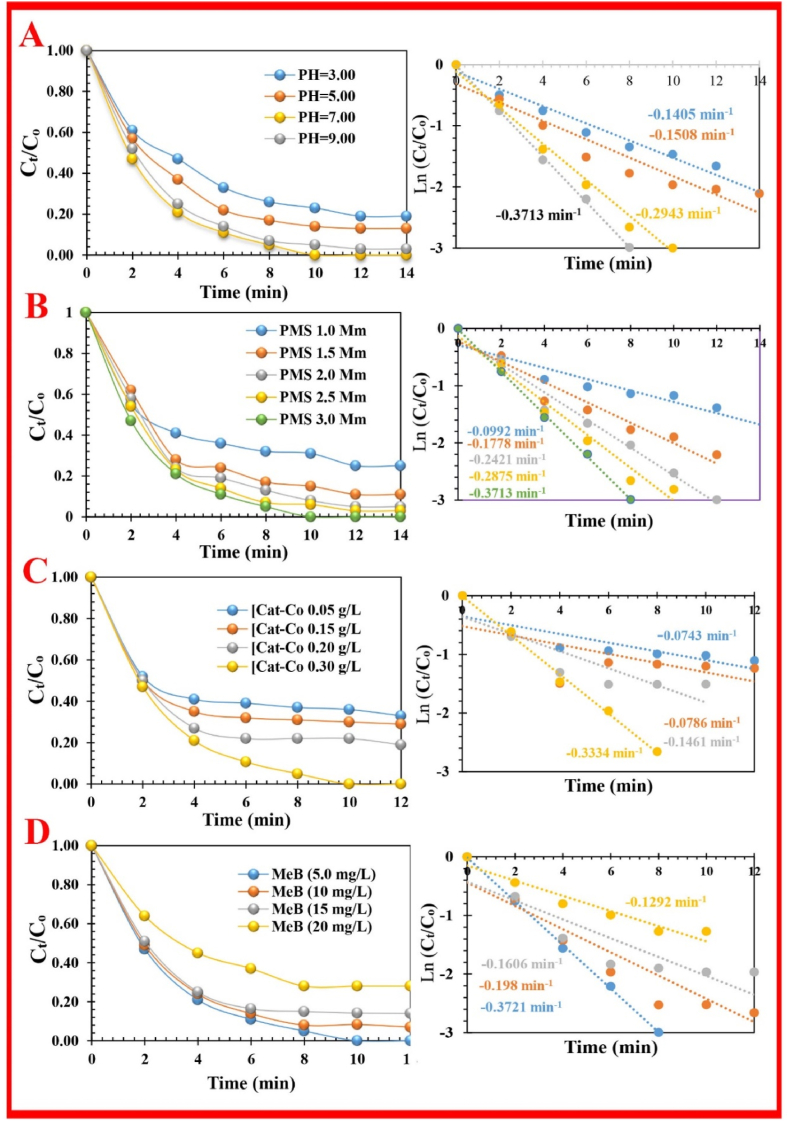
The effect of several parameters on degradation efficiency and the constant rate of MeB; (**A**) pH effect, (**B**) PMS dosage effect, (**C**) FS-(Am/g/Cs)@CoNP dosage effect, and (**D**) Initial MeB dosage effect.

**Fig. 5:**
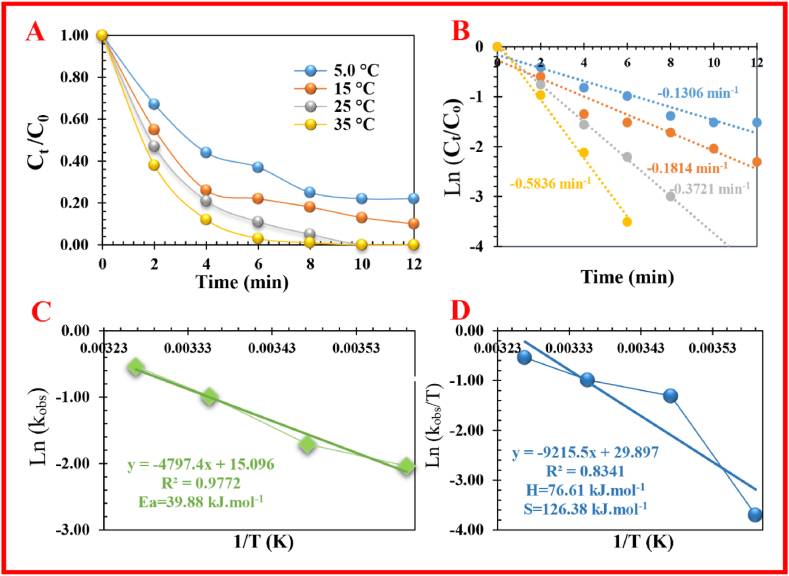
The effect of temperature on degradation efficiency (**A**), constant rate of MeB (**B**), and thermodynamic parameters (**C** and **D**).

To investigate the catalytic efficiency of Nano-biocomposite in MeB degradation, effective factors, including activator concentration (0.05< [Co-Cat]_0_< 0.30 g/L), reaction temperature (5–35 °C), PMS concentration (0.5 < [PMS]_0_ < 3.0 mM), the MeB concentration (5 < [MeB] _0_< 25 mg/L), time (0 < *t* < 12 min) and pH (3. 0 < pH < 9.0), were evaluated through UV spectroscopy at 664 nm (Fig. 3, Fig. 4, Fig. 5).

The low adsorption capacity of MeB dye (7.81 %) by FS-(Am/*g*/Cs)@CoNP may correspond to a high concentration of loaded Co and the compressed structure after the pyrolysis procedure. Additionally, these observations confirmed that the self-degradation rate of MeB without the PMS was *ca.*11.2 % with *k*_obs_ = 0.0119 min^−1^) (Fig. 3). The addition of FS-(Am/g/Cs)@CoNP enhanced the discoloration efficacy (and *k*_obs_) *ca*. 99.3 % (0.3703 min^−1^) within 12 min at room temperature. In the MeB/PMS system lacking FS-(Am/*g*/Cs)@CoNP as catalyst, the removal efficiency (and *k*_obs_) *ca*. 35.7 % (0.0387 min^−1^) of MeB was obtained in 8.0 min (Fig. 3). Against the homogenous Co^2+^ initiation process, the Co^2+^/Co^3+^ activation occurs on the surface of the FS-(Am/*g*/Cs)@CoNP.

The pH of the initial solution, by changing the surface, the solubility/species of the MeB dye, and the proportion of PMS, has a straight effect on the RE (Fig. 4A). The results revealed that the ideal degradation efficiency of MeB was achieved at neutral conditions. The rise of pH (3. 0 < pH < 9.0) was beneficial for the RE of MeB, because FS-(Am/g/Cs)@CoNP could more effectively activate the decomposition of PMS to produce a relatively high concentration of active radicals [37]. The results show that by increasing the pH (3.0–7.0), the RE of MeB on the PMS/FS-(Am/g/Cs)@CoNP system increased from 77 % (*k*_obs_ = 0.1405 min^_1^) to ∼100 % ((*k*_obs_ = 0.2943 min^_1^). Due to the remarkable increase in the repellant force at pH < 7.0, the positively charged surface disallowed the effective interaction of the cationic dye (pk_a_ = 3.14) with the PMS/FS-(Am/g/Cs)@CoNP system. At high alkaline solutions (>9.0), the subsequent reasons can slightly reduce the RE of MeB below the identical condition: store of OH^−^ and blocking the dynamic place of activator, scavenging off the SO_4_^●−^ by OH^−^ and generating ^●^OH, the produce of Co(OH)_2_ and then reduction of the catalytic action of the FS-(Am/g/Cs)@CoNP [38,39]. The outstanding RE of MeB in the PMS/FS-(Am/g/Cs)@CoNP system under acidic, neutral, and alkalescent conditions discloses the hopeful application possible for dye elimination in several conditions.

The PMS is the source for the generation of reactive species (*i.e.,* SO_4_^•-^ & ^•^OH) [37,40]. The RE (%) of dye showed a positive/negative dependence on the PMS concentration. With the increase in PMS dosage from 1.0 mM to 1.5, 2.0, 2.5, and 3.0 mM, the RE of MeB was increased from 0.1 mM (69 % with *k*_obs_ = 0.992 min^_1^) to 3.0 mM (∼100 % with *k*_obs_ = 0.3713 min^_1^) (Fig. 4B). Nevertheless, the RE of MeB was slightly enhanced, with further increasing from 1.0 mM to 3.0 mM. This might be attributed to the recombination of SO_4_^•-^ and ^•^OH and the sweeping reaction of reactive radicals by inordinate PMS [38].

As shown in Fig. 4D, the influence of the initial dosage of MeB dye on the RE was studied. The catalyst surface provides reaction sites for the discoloration of dye. The dual effect of FS-(Am/g/Cs)@CoNP dosage on the RE of MeB dye during the discoloration process was evaluated. According to Fig. 4C, the catalyst dosage had a remarkable effect on discoloration at the end of the treatment, where the RE increased from 64 % (at 0.05 g/L with *k*_obs_ = 0.0743 min^_1^) to ∼100 % (at 0.30 g/L with *k*_obs_ = 0.3334 min^_1^). Beyond and above this quantity (0.30 g/L), the RE decreased because of surface saturation and aggregation, respectively. The RE was reduced from ∼100 % to 72 % (*k*_obs_ = 0.3721 min^_1^ to *k*_obs_ = 0.1292 min^_1^) with the increase of the MeB dosage from 5.0 mg/L to 20 mg/L. At a high dosage of MeB (>15 mg/L), factors such as insufficiency of PMS compared to MeB dye molecules, block of the active site with excess dye, and production of intermediates can lead to decreased RE (Fig. 4D).

As the reaction temperature and time are the main factors in the favorable decoloration process, their effect was checked. The high temperature (5–25 °C) will result in a higher reaction rate between PMS and MeB dye in an endothermic process (Fig. 5). But, when increasing the temperature of the dye solution from 25 to 35 °C, a slight increase was observed in the reaction rate. The helpful temperature effect on removal efficiency (from 78.4 % to 99.8 %) and *k*_obs_ (from 0.1306 min^_1^ to 0.1814 min^_1^) in the variety of 5–25 °C for 14 min (Fig. 5), confirmed the endothermic nature of the removal process. It seems that the *k*_obs_ increased to 0.3721 min^_1^ by enhancing the temperature of the degradation system from 25 to 35 °C due, mainly, to the production of extra free radicals. Furthermore, as stated by the Arrhenius equation, the activation energy (*E*_a_) for MeB elimination in the PMS/FS-(Am/g/Cs)@CoNP system was realized to be 39.88 kJ mol^_1^ (R^2^ = 0.9772). Thus, it can be concluded that MeB degradation using FS-(Am/g/Cs)@CoNP can have adequate removal efficiency within 14 min at a temperature from 5 to 35 °C (Fig. 5).

As the discoloration process is an endothermic reaction, the RE (%) was heightened as the reaction temperature increased from 0 to 25 °C (Fig. 5) which caused an increase in the kinetic energy of the activator and PMS. These results can be associated with the improvement of the intensity of the transfer/collision of the reactants at high temperatures. The *E*_a_ (activation energy), DS^#^ (activation entropy), and DH^#^ (activation enthalpy) were calculated using the Arrhenius and Eyring equation [37,40]. The *E*_a_, ΔH^#^ and ΔS^#^ values of the catalytic removal procedure of MeB dye were obtained to be 39.88 kJ mol^−1^, 76.61 kJ mol^−1^ K^−1^, and 126.38 kJ mol^−1^, respectively. The favorable thermodynamic parameter and *E*_a_ of FS-(Am/g/Cs)@CoNP, make it a suitable activator for MeB degradation (Table 2, Table 3, Table 4).

**Table 2:** Comparison Entropy of FS-(Am/g/Cs)@CoNP in MeB degradation with other heterogeneous systems.

No	Catalytic system	ΔS (K. J mol^−^a)	Ref
1	QAMPSA/VPFe_3_O_4_^a^/H_2_O_2_	0.665	[37]
2	Pt_55_/Co_3_O_4_/H_2_O_2_	−93.20 ± 6.95	[38]
3	Nickel hydroxide/H_2_O_2_	0.077	[41]
4	NSC@S^2^/H_2_O_2_	−165.13 95	[42]
5	FS-(Am/g/Cs)@CoNP	126.38	***This work***

a QAMPSA/VP = uaternized diethylethanolamine cation joint with 2- acrylamido-2-methylpropane sulfonate-co vinylpyrrolidone.

**Table 3:** Comparison Activation Energy of FS-(Am/g/Cs)@CoNP in MeB degradation with other heterogeneous systems.

No	Catalytic system	Ea (K·J·mol^−1^)	Ref
1	QAMPSA/VPFe_3_O_4_^a^/H_2_O_2_	88.93	[37]
2	Pt_55_/Co_3_O_4_/H_2_O_2_	30.81 ± 1.15	[38]
3	Ferrocene/H_2_O_2_	82.71	[39]
4	FeCo_2_O_4_-N-C-400^b^/PMS	70.26	[40]
5	S-EAF^c^/H_2_O_2_	49.867	[43]
6	CoMn_30_Ce_10/_ H_2_O_2_	56.4	[44]
7	0.075-CoFe_2_O_4_/ZIF8/PMS	13.24	[45]
8	CuO/H_2_O_2_	75.80	[46]
9	CuO/H_2_O_2_	46.67	[47]
10	Fe_80_Si_1_P_10_C_9_ MSR^d^/H_2_O_2_	14.10	[48]
11	Fe_80_Si_1_P_10_C_9_ BMP^e^/H_2_O_2_	9.59	[48]
12	Fe_80_Si_1_P_10_C_9_ MAP^f^/H_2_O_2_	17.63	[48]
13	NPCs^g^700/PMS	48.38	[49]
14	Nickel hydroxide/H_2_O_2_	26.2	[41]
15	FS-(Am/g/Cs)@CoNP	39.88	***This work***

a QAMPSA/VP = uaternized diethylethanolamine cation mixed with 2- acrylamido-2-methylpropane sulfonate-co vinylpyrrolidone.

b 400 °C calcined nitrogen-containing carbon/FeCo_2_O_4_ composites.^3^ Peroxymonosulfate.

c Abundant electric arc furnace steel slag activated by sulfuric acid.

d Melt-spun ribbon.

e Ball milling powder.

f Multistageatomized powder.

g N-doped porous carbons.

**Table 4:** Comparison Entalpy of FS-(Am/g/Cs)@CoNP in MeB degradation with other heterogeneous catalytic systems.

No	Catalytic system	ΔH (kJ mol^−^a)	Ref
**1**	QAMPSA/VPFe_3_O_4_^a^/H_2_O_2_	86.30	[37]
**2**	Pt_55_/Co_3_O_4_/H_2_O_2_	51.33 ± 2.46	[38]
**3**	Nickel hydroxide/H_2_O_2_	−12.3	[41]
**4**	NSC@S^b^/H_2_O_2_	32.43	[42]
**5**	FS-(Am/g/Cs)@CoNP	76.61	***This work***

a QAMPSA/VP = uaternized diethylethanolamine cation mixed with 2- acrylamido-2-methylpropane sulfonate-co vinylpyrrolidone.

b SiO_2_-NH_2_-Cu(II)@SiO_2_.

**Scheme 2:**
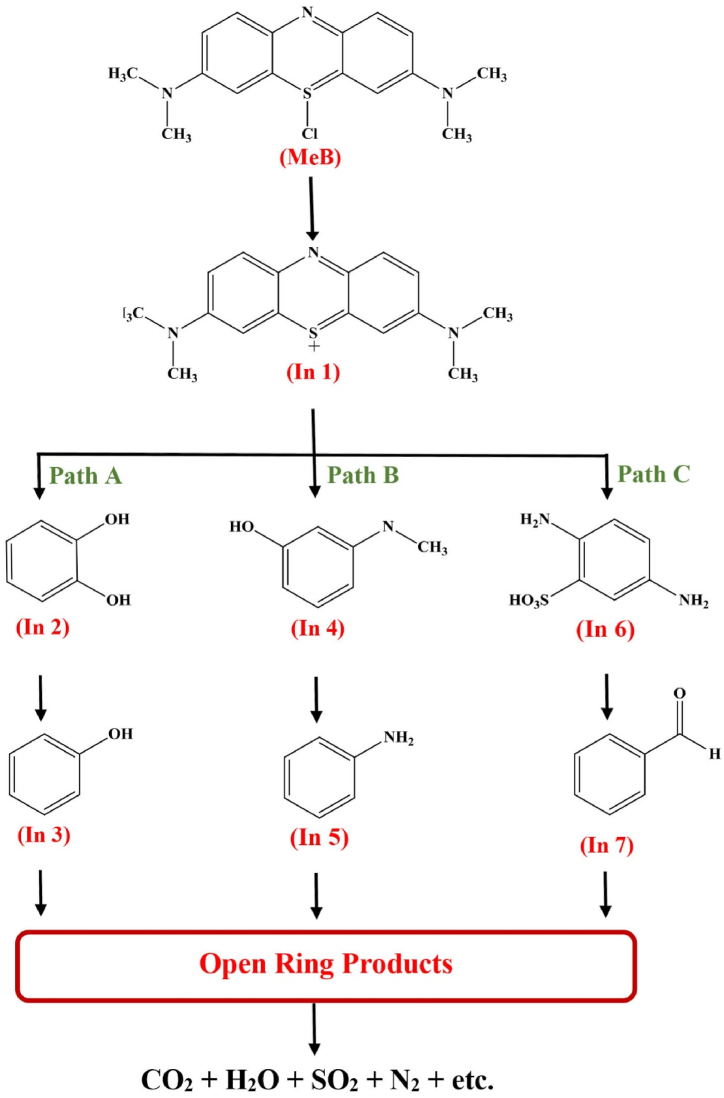
The proposed mechanism for MeB degradation by FS-(Am/g/Cs)@CoNP.

The time effect on RE of MeB dye was assessed at different time intervals, *e.g.,* 2, 4, 6, 8, and 10 and 12 min at room temperature. The increase in temperature and time caused an increase in the *RE* of MeB dye. Finally, the optimum temperature and time were found to be 25 °C and 10 min. Based on the obtained results, as mentioned above, the degradation mechanism of MeB by FS-(Am/g/Cs)@CoNP was suggested (Scheme 2).

**Fig. 6:**
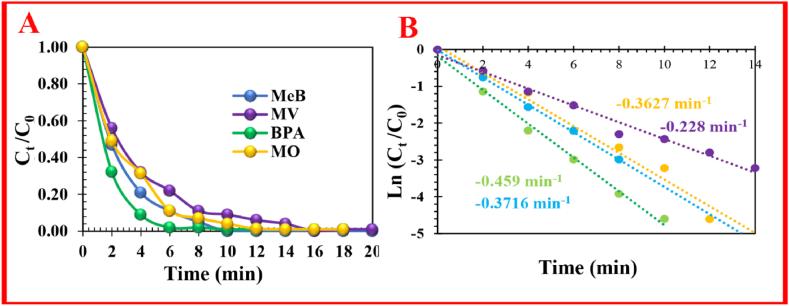
Degradation of diverse typical organics dyes, (A) removal efficiency, and (B) constant rate. Reaction condition: [FS-(Am/g/Cs)@CoNP]_o_ = 3.0 g/L, [pollutant]o = 5.0 mg/L, pH = 7.0, T = 25 °C, and [PMS]o = 3.0 mM)].

The applicability of the PMS/FS-(Am/*g*/Cs)@CoNP was examined based on its RE for diverse organic pollutants (*i.e*., methyl orange (MO), methylene violet (MV), and Bisphenol A (BPA)) as shown in Fig. 6. The degradation efficiency (and *k*_obs_) for MO, MV and BPA extended to 97.4 % (0.3627 min^−1^), 93.6 % (0.228 min^−1^) and 98.8 % (0.459 min^−1^) after 14 min at room temperature, respectively (Fig. 6A). The noticeable distinction in RE and constant rate is related to their dissimilar M_w_ (molecular weights) and chemical structures. These results illustrated that PMS/FS-(Am/*g*/Cs)@CoNP can be useful as an effectual activator for the high-performance degradation of numerous organic pollutants.

#### 3.2.2 Selective aerobic allylic oxidation of cyclohexene

**Fig. 7:**
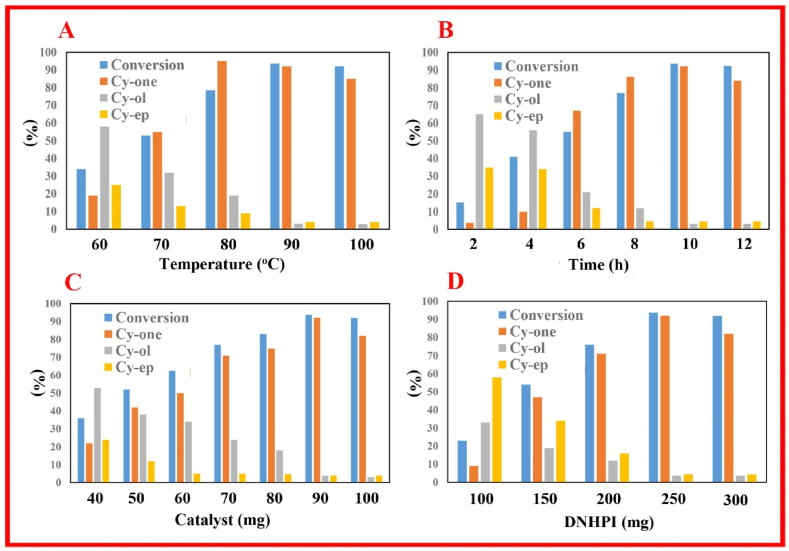
Catalytic oxidation of Cy at different conditions; (**A**) temperature effect, (**B**) time effect, (**C**) amount of nano-biocomposite effect, and (**D**) DNHPI dosage effect. Reaction Condition: FS-(Am/g/Cs)@CoNP = 90 mg, DNHPI = 250 mg, *t* = 10 h, HAc/H_2_O = 1.5/1.0 v/v under O_2_ bubbling.

As it produces three extensively utilized compounds, namely 2-cyclohexene-ol (Cy-*ol*), cyclohexene oxide (Cy-*ep*), and 2-cyclohexene-one (Cy-*one*), the selective oxidation of cyclohexene (Cy) is of enormous relevance to various sectors (including agrochemical, pharmaceutical, and fragrance) [50]. Fig. 7 shows how important variables (such as catalyst loading, time and temperature of the reaction, and amount of oxidant) affect the selective allylic oxidation of Cy. The reaction temperature has been varied from 60 °C to 100 °C to examine the role of temperature on the conversion of Cy to produce selective oxidation products. It was utilizing the FS-(Am/*g*/Cs)@CoNP from the current study that performed the best. The Cy conversion and Cy-*one*'s selectivity have increased linearly with reaction temperature, as shown in Fig. 7A. The catalyst exhibits 100 % overall selectivity to Cy-one and ∼98 % conversion. However, the reaction temperature was raised further, from 60 °C to 90 °C. The consequence of reaction time on the oxidation of Cy with DNHPI is shown (2–12 h) in Fig. 7B. The FS-(Am/*g*/Cs)@CoNP displays 100 % overall conversion and selectivity to Cy-one in 10 h. The yield and selectivity of Cy-one increased somewhat by passing the time from 2 h to 10 h; after that, the profit decreased (12 h). This showed that after 10 h of reaction time, the Cy oxidation process achieved a condition of chemical equilibrium.

**Scheme 3:**
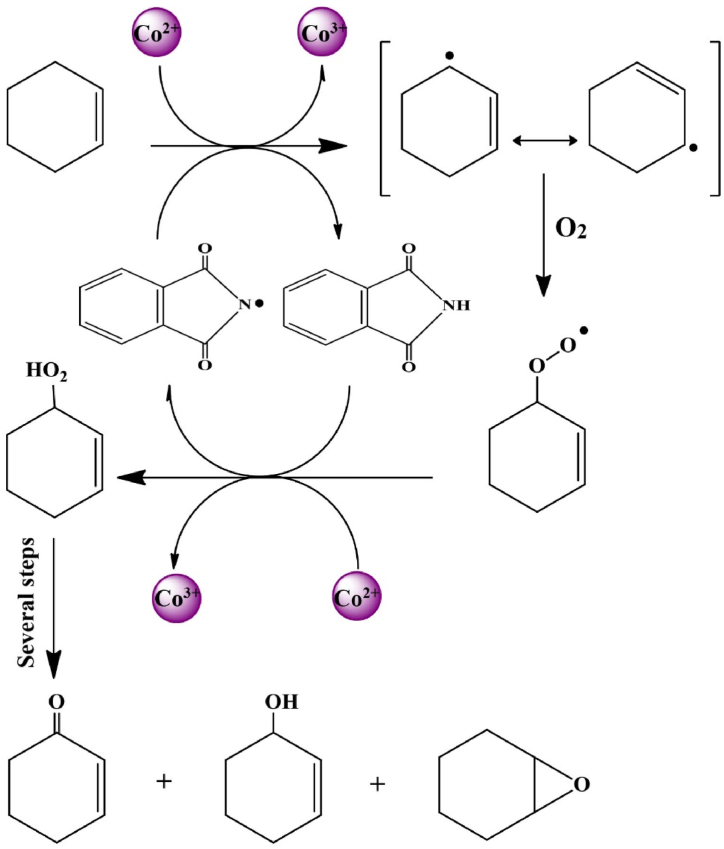
The proposed mechanism of selective aerobic allylic oxidation of cyclohexene by FS-(Am/g/Cs)@CoNPs.

Fig. 7C depicts how the amount of catalyst affected the conversion and selectivity. With 40–70 mg of catalyst, Cy-*one* selectivity and conversion are substantially lower than with 90 mg of catalyst. The Cy-*one* yield and selectivity are reduced when the catalyst concentration is more than 90 mg. The experiment's findings suggest that having a lower than 80 mg catalyst can make other compound production easier, such as Cy-*ol* and Cy-*ep* (Scheme 3). The recommended catalyst dose is 90 mg.

DNHPI were tested (100–300 mg) to see how they affected the oxidation of Cy. The reactions were conducted under O_2_ pressure at 90 °C. Fig. 7D shows the product selectivity and Cy conversion at various oxidant concentrations. The findings demonstrated that raising the DNHPI concentration considerably enhanced the conversion of Cy. The amount of DNHPI was raised from 100 mg to 250 mg, and this did result in a higher rate of Cy conversion. So the best concentration of DNHPI for the oxidation of Cy is 250 mg. A possible catalytic mechanism that explains the formation of Cy-*ol*, Cy-*ep* and Cy-*one*, is shown in Scheme 3. In this catalytic process, Cy-*ep* and Cy-*one* were not observed and the nano-biocomposite exhibits 100 % selectivity to Cy-one. High selectivity to Cy-one is one of the characteristic features of FS-(Am/*g*/Cs)@CoNP compared to other catalysts (Table S2).

### 3.3 Stability, leaching, and long term study of nano-biocomposite

**Fig. 8:**
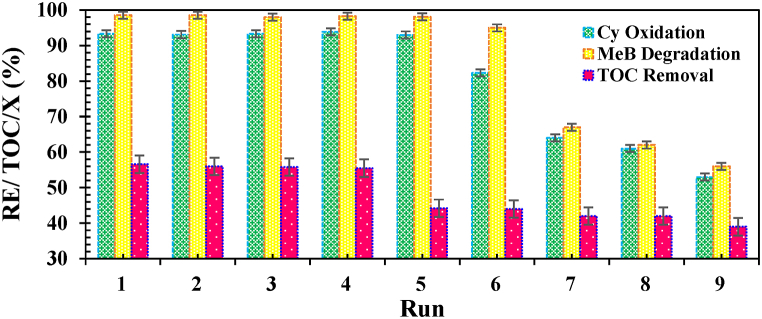
Long-term stability in degradation of MeB (**A**) and aerobic oxidation of cyclohexene (**B**) and TOC Removal efficiency of MeB (**C**). Reaction Condition:**(A)** [FS-(Am/g/Cs)@CoNP] = 0.30 g/L, [MeB] = 5.0 mg/L, pH = 7.0, T = 25 °C, [PMS] = 3.0 mM)].**(B)**[[FS-(Am/g/Cs)@CoNP] = 90.0 mg, [Cy] = 9.0 mM, T = 90 °C, DNHPI = 250 mg, HAc/H_2_O = 1.5/1.0 v/v under O_2_ bubbling].

The reusability of *nano-biocomposite* is a principal factor due to their industrial potential. The recyclability of FS-(Am/g/Cs)@CoNP was investigated for the repeated degradation of MeB dye and selective allylic oxidation under optimal conditions. An external magnet was applied to isolate the FS-(Am/*g*/Cs)@CoNP from the degradation and aerobic oxidation system and then reused 5 and 6 times in the fresh reactions, respectively (Fig. 8). Based on the results obtained from FT-IR, XRD (Fig. S1 and Fig. S2), microscopic analysis, the leaching test, and ICP-OES detection limit [51], we were unsuccessful to discovery the CoNPs in the reaction solution. Also, no destruction of chemical structure, which approves the chemical and mechanical stability of FS-(Am/*g*/Cs)@CoNP and the captured CoNPsin the 3D-organic framework, was observed (Fig. S1 and Fig. S2). Results illustrate a slight decrease in the catalytic performance of the FS-(Am/g/Cs)@CoNP with the increase in recycle times, which may result from the aggregation and loss of active center and block/oxidation of the FS-(Am/*g*/Cs)@CoNP surface. These results confirmed that the FS-(Am/g/Cs)@CoNP has acceptable catalytic stability during the activation of PMS and oxygen molecules.

**Fig. 9:**
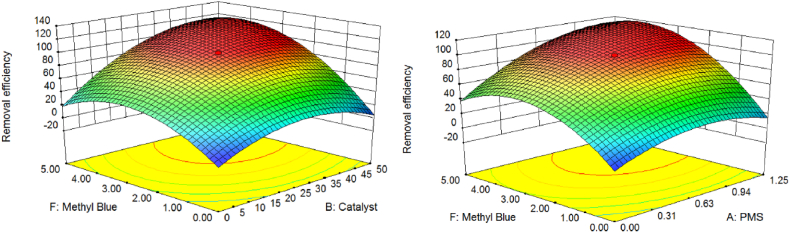
The combined effect of catalyst dosage and PMS on removal efficiency of MeB.

Herein, the association between response (RE (%)) and fourth independent factors (temperature, time, catalyst dosage, and PMS concentration) was studied using the response surface method (RSM) (Table S4). The comparison of the predicted and experimental values with the RSM model indicates a statistically noteworthy difference (p < 0.05). As a consequence, the obtained experimental results were in contract with the conditions. Fig. 9 exhibits the joint effect of nano-biocomposite amount and temperature on RE (%). The exact analysis of the four variables determined that the greatest critical factor was temperature, with the amount of catalyst being the second most important factor on the RE (%).

### 3.4 Comparative study

The compressive study on the catalytic efficiency of FS-(Am/g/Cs)@CoNPs compare to other catalytic systems in MeB decolorization and oxidation of cyclohexene, were done. By calculating the cost of our method with another MB degradation process (S3, Table S3), and owning the sensible cost and recyclable capability, we can present the PMS + FS (Am/*g*/Cs)@CoNP system as an operational system (Table S3). Also, comparisons of some catalytic systems for MeB degradation with the PMS + FS-(Am/*g*/Cs)@CoNP system (S4) reveal that it is a pioneer in terms of eco-friendliness, biocompatible shell, and solubility in polar and nonpolar conditions, magnetic properties, simple recovery, operative coating of chitosan with amino/glutaraldehyde groups, facile route and use commercially accessible material for production, thermal/chemical stability, rapid removal process, usability in wide ranges of pH and temperature, use PMS as a green oxidant, excellent removal efficiency. Thereby, the PMS + FS-(Am/*g*/Cs)@CoNP system is very appropriate for the degradation of MeB (Table S1) and other pollutant dyes.

To investigate the efficiency and profitableness of this protocol, the results were compared with other oxidation reaction in order to evaluate the advantages and disadvantages of this catalytic system (Table 3). This comparison showed that the our catalytic oxidation reaction has advantages in terms of solid nature of catalyst, inexpensive and stability of catalyst, short reaction time, high conversion and selectively, use of O_2_ as an eco-friendly oxidant and widely used in catalytic oxidation reaction. Therefore, this protocol could be introduced for practical organic synthesis.

## 4 Conclusion

In this study, we tried to demonstrate that the as-prepared FS-(Am/g/Cs)@CoNP has excellent efficiency in the activation of PMS/O_2_. In the preparation of networked Co(II)-Chitosan-*g*-APAS magnetic beads, glutaraldehyde not only serves as a cross-linking agent but also links the amine groups and/or chitosan through primary and/or secondary amine groups (either *N*-chain or N-terminal) by N-amine and/or Schiff base linkage to chitosan. Catalytic studies have shown that FS-(Am/g/Cs)@CoNP has high activity, selectivity, stability, and reusability in the degradation of organic dyes and selective allylic oxidation of Cyclohexene with PMS and molecular oxygen, respectively in a relatively short response time at room temperature. The nano-biocomposite exhibited potential for applications in green environmental governance. The catalytic process involves several highlights such as the heterogeneous nature of nano-biocomposite, environmental-friendly, mild reaction conditions, and excellent efficiency in the absence of any reducing agents and promotors. Additionally, costly, the negligible effect of organic and/or inorganic compounds and suitability for real environments and high recovery efficiency were evaluated based on economic, interface, and Long-term study, respectively.

## CRediT authorship contribution statement

**Maryam Lotfi:** Visualization, Software, Data curation. **Alireza Faraji:** Writing – review & editing, Writing – original draft, Validation, Software, Methodology, Formal analysis. **Fatemeh Ashouri:** Writing – original draft, Supervision, Project administration, Methodology, Investigation, Conceptualization.

## Declaration of competing interest

The authors declare the following financial interests/personal relationships which may be considered as potential competing interests.
