# Supplementary material for: Modified magnetic Nano-Biocomposite as friendly environmental catalyst for rapid degradation of organic dyes and selective aerobic oxidation of cyclohexene through advance oxidation process
Source: Heliyon. 2024 Sep 26;10(19):e38453. doi: 10.1016/j.heliyon.2024.e38453 (PMC11472097; doi:10.1016/j.heliyon.2024.e38453)
Supplement: Multimedia component 1 [file mmc1.docx]

# **Supplementary Data**

# **Modified Magnetic Nano-biocomposite as Friendly Environmental Catalyst for Rapid Degradation of Organic Dyes and Selective Aerobic Oxidation of Cyclohexene through Advance Oxidation Process**

Maryam Lotfi^a^, Alireza Faraji^b,c^ Fatemeh Ashouri^d,^*

**^a^***Active Pharmaceutical Ingredients Research Center, Tehran Medical Sciences, Islamic Azad University, Tehran, Iran.*

***^b^****Department of Organic Chemistry, Faculty of Pharmaceutical Chemistry, Tehran Medical Sciences, Islamic Azad University, Tehran, Iran.*

**^c^**Nutrition and Food Sciences Research Center, Tehran Medical Sciences, Islamic Azad University, Tehran, Iran.

**^d^***Department of Applied Chemistry, Faculty of Pharmaceutical Chemistry, Tehran Medical Sciences, Islamic Azad University, Tehran, Iran.*

*Corresponding author. Tel.: +98 21 22640051; fax: +98 21 22600099.*

*E-mail address:* f.ashouri@iaups.ac.ir

**S1. Response Surface Method**

Herein, the association between response (RE (%)) and fourth independent factors (temperature, time, catalyst dosage, and PMS concentration) was studied using the response surface method (RSM). The experimental plan involved the response surface statistical method and the use of the Box-Behnken design. The RSM method was used for the decolonization of MeB via FS-(Am/g/Cs)@CoNP because this combination resulted in high RE (%). Optimization of handling to maximize the RE (%) was done using the RSM model. (FS-(Am/g/Cs)@CoNP =25 mg, PMS=0.63 mM, *t*=60 min, T=35 °C, MeB=2.50 mg/L pH=7.50). Moreover, the response model equations, and ANOVA (analysis of variance, Table. S4) for the response surface quadratic model were accomplished. The coefficient of determination (R^2^) and Adjusted-R^2^ calculated from the validation data for RSM models were 0.9641. According to coded and real parameters, the second-order RE (%) for predicting the catalytic decolorization of MeB is as follows:

Removal efficiency (%) = +100.00 +22.52 * A +27.50 * B +7.99 * C +34.20 * F +9.25 * A *B +14.51 * A *F +20.38 * B *F -20.29 * A^2^ -29.33 * B^2^ -10.15 * C^2^ -9.63 * D^2^ -38.40 * F^2^

where A, B, C, D, E, and F are the independent parameters of PMS dosage, FS-(Am/g/Cs)@CoNP loading, time, temperature, pH, and MeB dose, and RE (%) is the response factor in the peak area. The basis for the response surface quadratic model's analysis of variance (ANOVA). The FS-(Am/g/Cs)@CoNP factors could be represented by the model, according to the R^2^ and Adjusted-R^2^ values of 0.9641 and 0.9268.

**S2. Economic study on Methyl blue degradation process**

By calculating the cost of our method with another degradation process*,* the economic feasibility of MeB degradation using a designed Nano-biocomposite was examined. Regardless of energy consumption price, the construction of FS-(Am/g/Cs)@CoNP is the cost part of the process. As shown in Table 3S, the price for discoloration of MeB in the PMS+FS-(Am/g/Cs)@CoNP system is 246$ /m^3^. This is while the cost of PS+thermal (44.41 $ /m^3^) [71], PS+ultraviolet (0.176 $ /m^3^) [71] system and, chemical systems (PS+γ-Fe_2_O_3_-CeO_2_= 0.106$/m^3^ & PS+Fe^2+^ = 0.106$ /m^3^) is not very high. For single-use, the charge of the degradation process was much higher than those of the PS+thermal (44.41 $ /m^3^) [71], PS+ultraviolet (0.176 $ /m^3^) [71], and chemical systems (PS+γ-Fe_2_O_3_-CeO_2_= 0.106$/m^3^ & PS+Fe^2+^ = 0.106$ /m^3^) [72] (Table S3). Nevertheless, the FS-(Am/g/Cs)@CoNP had high reusability (Fig. 9), and the charge for discoloration of a single-use was estimated to be about 49.2 $/m^3^.

**S3. Comparison study on the Methyl blue degradation process**

The comparison of some catalytic systems for MeB decolorization and oxidation of cyclohexene, with accent on the last few years, is gathered in Table 1S and Table 2S, respectively. In the case of degradation of MeB, PMS+FS-(Am/g/Cs)@CoNP system has notable merits. As shown in Table S2, for selective allylic oxidation, the FS-(Am/g/Cs)@CoNP eco-friendly catalyst has notable advantages in terms of heterogeneous nature based on bio-magnetic properties and comprehensive separation, low cost, simplicity in production and application, need for low temperature and petite time, great stability, use O_2_ as a greener and waste-avoiding oxidant, safe and usual solvent, and brilliant catalytic performance. Thus, this process is very suitable for practical application (Table S2).

**Table S1.** Comparison of catalytic activity of FS-(Am/g/Cs)@CoNP with various catalytic systems in degradation of MeB reported in the literature.

| *NO* | **Catalytic System** | **t**  **(min)** | **Oxidant**  **[c]** | **Efficiency**  **(%)** | **pH** | **Ref** |
| --- | --- | --- | --- | --- | --- | --- |
| 1 | Co_3_O_4_/CoO [0.6 g/L] | 40 | NaHSO_3_[2 g/L] | 90.7 | 6.38 | [1] |
| 2 | Ce-doped UiO-67-400^1^[1 g/L] | 30 | H_2_O_2_[7 mmol/L] | 94.1 | 3 | [2] |
| 4 | Mg/Co(OH)_2_[1 g/L] | 120 | H_2_O_2_[10 mg/L] | 99.62 | >7 | [3] |
| 5 | NbCeO_x_^2^[20 mg] | 60 | H_2_O_2_[200 µL] | 83 | 5.1 | [4] |
| 6 | FeCo_2_O_4_-N-C-400^3^[0.010 g] | 10 | PMS[0.5 g/L] | 100 | Natural | [5] |
| 7 | CNTs/FeS_2_^4^[0.2 g] | 25 | H_2_O_2_/NH_2_OH[200 µL/60 mg] | 99.5 | 3 | [6] |
| 8 | sFe_3_O_4_-NRs^5^[5 mg] | 360 | H_2_O_2_[1 ml] | 100 | 3-11 | [7] |
| 9 | CoO/NaHSO_3_[0.6 g/L] | 6 | NaHSO_3_[2 g/L] | 99.4 | 3/12 | [8] |
| 10 | MIL-88A(Fe_1_Zn_1_)^6^[1 g/L] | 40 | H_2_O_2_[2.5 ml/L] | 96.15 | 3 | [9] |
| 11 | MgO[0.4 g/L] | 20 | PMS[0.8 g/L] | 99.4 | 7 | [10] |
| 12 | CoFe_2_O_4_/ZIF-8^7^[0.05 g/L] | 60 | PMS[0.3 g/L] | 97.9 | 6.3 | [11] |
| 13 | MnCo_2_O_4.5_[20 mg/L] | 25 | PMS[500 mg/L] | 100 | No pH | [12] |
| 14 | hematite/ZVI^8^[69 mg/L] | 60 | H_2_O_2_[5 mM] | 100 | 3 | [13] |
| 15 | MoS_2_-Fe_3_O_4_MNPs^9^[0.05 g/L] | 30 | H_2_O_2_[50 mmol/L] | 100 | 3 | [14] |
| 16 | CuO[20 mg] | 30 | H_2_O_2_[2 ml] | 100 | 5.6 | [15] |
| 17 | Fe_3_O_4_@PDA-MnO_2_^10^[5 mg] | 240 | H_2_O_2_[5 ml] | 97.36 | 3 | [16] |
| 18 | Cu@Co-MOFs-3^11^[0.1 g/L] | 30 | PMS/ PDS^12^[2 mM] | 100 | 11 | [17] |
| 19 | Fe*x*O*y*/N-GN/CNTs^13^[0.4 g/L] | 12 | PMS[2 mmol/L] | 98.75 | 7.62 | [18] |
| 20 | CoMoO_4_/AC^14^[0.1 g/L] | 60 | PMS[2 mM] | 90 | No pH | [19] |
| 21 | Fe_3_O_4_@ZIF-8^15^[1 g/L] | 30 | SPC^16^[4 mmol/L] | > 90 | 3 | [20] |
| 22 | SUST/Fe-BTC^17^[0.6 g/L] | 60 | H_2_O_2_[7.4 mmol/L] | 95 | 4 | [21] |
| 23 | Nb_2_O_5_^18^[0.1 g] | 120 | H_2_O_2_[5 ml] | 100 | 5.2 | [22] |
| 24 | SBMC^19^[0.4 g/L] | 3 | H_2_O_2_[39 mmol/L] | 98.56 | 3 | [23] |
| 25 | magnesium porphyrin complex^20^[5 mg] | 25 | H_2_O_2_[4 ml/L] | 82 | 6 | [24] |
| 26 | Fe@S-1^21^[0.5 g/L] | 30 | H_2_O_2_[4 mmol/L] | 100 | 2 | [25] |
| 27 | MgCoAl-LDH^22^[0.02 g/L] | 40 | PMS[1 mM] | 100 | 6 | [26] |
| 28 | CuFe_2_O_4_@ZIF-67^23^[75 mg/L] | 30 | PMS[125 mg/L] | 98.9 | 4 | [27] |
| 29 | NPCs^24^[0.1 g/L] | 30 | PMS[2 mM] | 99.15 | 5.76 | [28] |
| 30 | CuCo-ZIF^25^[50 mg/L] | 100 | H_2_O_2_[0.1 mg/L] | 98 | 3 | [29] |
| 31 | CU-Cy^26^[0.5 mg/mL] | 6 | H_2_O_2_[50 mM] | 98 | Natural | [30] |
| 32 | NbCo-MOF^27^[0.01 g] | 30 | PMS[0.3 g/L] | 100 | 6.02 | [31] |
| 33 | NSC@S^28^[0.03 g] | 30 | H_2_O_2_[0.2 M] | 93.8 | 7 | [32] |
| 34 | FeMnO_3_[0.2 g/L] | 60 | PMS[2 g/L] | 98 | 6.7 | [33] |
| 35 | TP-SF/Fe^29^[0.1 g] | 40 | H_2_O_2_[0.1 mmol/L] | 98 | 3/11 | [34] |
| 36 | FS-(Am/g/Cs)@CoNP[0.3 g/L] | 10 | PMS[3.0 mM ] | ~100 | 7 | This work |

^1^ Ce-doped MOF, calcination temperature of catalyst 400 °C, MOF=Metal–organic framework.

^2^ Mixed Niobium-Cerium Oxide.

^3^ 400 °C calcined Nitrogen-containing Carbon/FeCo_2_O_4_ composites.

^4^ CNTs/FeS_2=_Carbon Nanotubes/pyrite nanocomposite.

^5^ Supermicropores ferrimagnetic Fe_3_O_4_ Nano Rings.

^6^ ZnO doped MIL-88A (a Metal-Organic Framework based on Fe (III) trimers, MIL=Matériaux del′Institut Lavoisier.

^7^ Cobalt ferrite/ Zeolite Imidazolate Framework-8.

^8^ ZVI=Zero-Valent Iron.

^9^ MNPs=Magnetic Nano Particles.

^10^ PDA=Polydopamine.

^11^ MOF= Metal Organic Framework.

^12^ PDS=Peroxydisulfate.

^13^ Three-Dimensional Multifunctional FexOy/N-GN/CNTs, N-GN=Nitrogen-doped Graphene, CNTs=Carbon Nanotubes.

^14^ Activated Carbon-Supported Cobalt Molybdate Composite.

^15^ ZIF= Zeolite Imidazolate Framework.

^16^ SPC=Sodium Per Carbonate

^17^ Sustainable MOF Materials Basolite F300-like semi-amorphous Fe- BTC, BTC=1,3,5-Benzene Tri Carboxylate.

^18^ Niobium(V) Oxide.

^19^ Fenton Sludge was converted into Magnetic Sludge-based Biochar.

^20^ Magnesium (II) Porphyrin with the ligand Hexamethylenetetramine.

^21^ S-1 Zeolite encapsulated Fe nanocatalyst.

^22^ LDH=Layered Double Hydroxide.

^23^ ZIF-67= Zeolite Imidazolate Framework-67.

^24^ NPCs=N-doped Porous Carbons.

^25^ ZIF= Zeolite Imidazolate Framework.

^26^ Copper-Cysteamine.

^27^ MOF= Metal Organic Framework.

^28^ NSC@S =SiO_2_-NH_2_-Cu(II)@SiO_2_ nanoreactors.

^29^ The modified Silk Fabrics.

**Table S2.** Comparison of catalytic activity of FS-(Am/g/Cs)@CoNP with various catalytic systems in aerobic oxidation of cyclohexene reported in the literature.

| No. | Catalytic System | *X* (%) |  | Selectivity (%)  Cy-*ol* Cy-*one* Cy-*ep Other* | | | | [Ref] | | |
| --- | --- | --- | --- | --- | --- | --- | --- | --- | --- | --- |
| 1 | Co(II)-L^1^@nano-SiO_2_[20 mg]/ 75^◦^C/ 8 h/ Acetonitrile[5 ml]/ O_2_[0.2 MPa] | 61 |  | 9.3 51.9 | 7.4 | 31.4 | | [35] | | |
| 2 | CoOx/SiO_2_[20 mg]/ 60^◦^C/ 15h/ free/ O_2_ | 58 |  | 44 43 | 13 |  | | [36] | | |
| 3 | LaCoODA^2^[0.01 mmol]/ 75^◦^C/ 24h/ free/ O_2_[1 bar] | 85 |  | 25 75 | - |  | | [37] | | |
| 4 | rGO^3^/Fe_3_O_4_[0.05 g]/ 70^◦^C/ 6h/ Acetonitrile[5 ml]/ H_2_O_2_[10 mmol] | 75.3 |  |  |  | 81 | | [38] | | |
| 5 | SGU-29^4^[0.01g]/ 70^◦^C/ 12h/ Acetonitrile [4.8 g]/ O_2_[1 bar] | 42.2 |  | 23.3 47.7 |  | 34.4 | | [39] | | |
| 6 | Co_3_O_4_[0.1 g]/ 70^◦^C/ 6h/ Heptane[20 ml]/ TBHP^5^[5.5 ml] | 78 |  | 43 41 |  | 16 | | [40] | | |
| 7 | HA/HCl-18 h/Co^2+^-2:1^6^[40 mg]/ 75^◦^C/ 18h/ free/ O_2_ | 58.30 |  | 39.43 54.60 | 0.27 | 5.70 | | [41] | | |
| 8 | Ru/Ti-PILCs^7^[0.1 g]/ 70^◦^C/ 6h/ Heptane[25 ml]/ TBHP[5.5 ml] | 59 |  | 13 87 |  |  | | [42] | | |
| 9 | 5MoCT^8^[100 mg]/ 80^◦^C/ 10h/ Dichloromethane[5 ml]/ TBHP[20 mmol] | 100 |  |  | 91 |  | | [43] | | |
| 10 | MoSi-500^9^[20 mg]/ 65^◦^C/ 2h/ Toluene[10 cm^-3^]/ cmOOH^10^[4.9 mmol] | 86 |  | 9 | 90 | 1 | | [44] | | |
| 11 | L2-Co(II)^11^[0.1 g]/100^◦^C/ 1h/ Acetonitrile[5 ml]/ H_2_O_2_[4 mmol] | 46.20 |  | 64.29 15.04 |  | 0.04 | | [45] | | |
| 12 | SBA.15. DAFO.Pd(II)^12^[0.06 g]/ 60^◦^C/ 12h/ Acetonitrile[6 ml]/ H_2_O_2_[2 ml] | 89.3 |  | 9.1 16.3 |  | 60.1 | | [46] | | |
| 13 | Co-POM-octyl-NH_3_-SBA15^13^[20 mg]/ 65^◦^C/ 3h/ Acetonitrile[5 ml]/ H_2_O_2_[18 mmol] | 83.8 |  | 0.8 1.6 | 92.8 | 4.8 | | [47] | | |
| 14 | GO-Cu-L^14^[40 mg]/ 75^◦^C/ 5h/ Acetonitrile[6 ml]/ H_2_O_2_[2 ml] | 100 |  |  | 100 |  | | [48] | | |
| 15 | Rh-TUD-1^15^[0.4 g]/ 60^◦^C/ 1h/ free/ H_2_[5 atm] | 100 |  |  | - | 100 | | [49] | | |
| 16 | p(APTMACl)/GO-Pd^16^[0.097 mmol]/ 60^◦^C/ 7h/ Acetonitrile[5 ml]/ O_2_ | 78 |  |  | > 99 |  | | [50] | | |
| 17 | Co/N:C^17^[10 mg]/ 70^◦^C/ 16h/ Acetonitrile[15 ml] /O_2_[10 bar] | 80 |  | 6 38 | 6 | 50 | | [51] | | |
| 18 | Co_15_PW_12_O_40_[0.2 g]/70^◦^C/ 4h/ Acetonitrile[5 ml]/H_2_O_2_,CO_2_[10 ml, 0.5 MPa] | 70.5 |  | 21.6 49.6 |  |  | 11.1 | | [52] |  |
| 19 | Ti-Zr-Co[20 mg]/ 120^◦^C/ 12h/ Acetonitrile[20 ml]/ O_2_[2 MPa] | 97.5 |  | 3.7 49.5 |  | 46.7 | | [53] | | |
| 20 | CoTPPS/Ni-Al /LDH_3.0_^18^[0,002 mmol]/ 25^◦^C/ 2h/ Acetonitrile[4 ml]/ O_2_ | 99 |  |  | 92 |  | | [54] | | |
| 22 | PS‐[CH2{Cu (sal‐tch) Cl}2]^19^[0.040g]/70^◦^C/ 6h/ Methanol[10 ml]/ H_2_O_2_[20 mmol] | 82.9 |  | 13.26 46.93 | 16.12 | 23.46 | | [55] | | |
| 23 | MG@NSal-Co^20^[3 mg]/ 70^◦^C/ 12h/ free/ O_2_ | 46.8 |  | 8.7 77.2 | 5.3 | 8.8 | | [5] | | |
| 24 | AuFeC[0.1 g]/ 80^◦^C/ 14h/ n-heptane[75 ml]/ O_2_[6 bar] | ≈50 |  | 9 10 |  | 81 | | [57] | | |
| 25 | MnPC^21^[1 µmol]/ 23^◦^C/ 4h/ Dichloromethane[4 ml]/ O_2_ | >99 |  |  | 92 |  | | [58] | | |
| 26 | GNPs/TChD^22^[50 mg]/ 80^◦^C/ 8h/ free/ O_2_[10 bar] | 87 |  | 70 |  |  | | [59] | | |
| 27 | Ru/TiO_2_ NPs^23^[25 mg, 28 μmol Ru]/ 75^◦^C/ 4.5h/free/ O_2_[4 bar] | 95 |  | 11 80 | 7 | 2 | | [60] | | |
| 28 | MoCeNR^24^[0.05 g]/ 80^◦^C/ 2h/ Toluene[2 ml]/ TBHP[0.6 ml] | 98.9 |  | 0.9 1.8 | 97.3 |  | | [61] | | |
| 29 | AgCN^25^[1 mg]/ 60^◦^C/ 20h/ Acetonitrile[2.5 ml]/ H_2_O_2_[0.5 ml] | ~100 |  |  | 76 |  | | [62] | | |
| 30 | Mo–Ti–SBA-15-(6.5)^26^[25 mg]/ 80^◦^C/ 4h/ n-octane[1.25 mmol]/ TBHP[5 mmol] | 88 |  |  | 98 |  | | [63] | | |
| 31 | SPDVB^27^[10 mg]/ 75^◦^C/ 24h/ free/H_2_O_2_[1.25 ml] | 97.22 |  | 0.94 2 | 24 | 95.7 | | [64] | | |
| 32 | PW_4_/CNTs^28^[10-30 mg]/ 50^◦^C/ 4h/DMC^29^[1 ml]/ H_2_O_2_[0.1 M] | 66 |  |  | 79 |  | | [65] | | |
| 33 | Ti-PMO-S10^30^[0.1 g]/ 70^◦^C/ 24h/ Acetonitrile[15 g]/ TBHP[13 mmol] | 30.45 |  | 3.2 2.4 | 98.9 | 0.5 | | [66] | | |
| 34 | PVDA1-PMo^31^[0.1 g]]/ 130^◦^C/ 6h/ Acetonitrile[10 ml] O_2_[0.6 MPa] | 85.7 |  | 51.9 |  |  | | [67] | | |
| 35 | SiO_2_/Al_2_O_3_-APTMS-BPK-Mn^32^[0.1 g]/ 120^◦^C/ 24h / acetic acid/benzonitril[5 cm^3^]/ NHPI[15%mol]/ O_2_ | 97 |  | 99.9 | trace |  | | [68] | | |
| 36 | SiO_2_/Al_2_O_3_-APTMS-BPK-Co [0.1 g]/ 100^◦^C/ 24h / acetic acid/benzonitril[5 cm^3^]/ NHPI[15%mol]/ O_2_ | 93 |  | 99.9 | trace |  | | [69] | | |
| 37 | FNC^33^[5 mg]/ 120^◦^C/ 24h / free/ TBHP[9 mmol] | 39 |  | 96 |  |  | | [70] | | |
| 38 | FS(Am/g/Cs)@CoNP[90mg]/90^◦^C/10h/acetic acid/DNHPI[250mg] | ? |  | ? | ? | ? | | **This work** | | |
|  |  |  |  |  |  |  | |  | | |

^1^ L=N-(3-trioxysilyl-propyl)-o-vanillin imine.

^2^ ODA=Oxydiacetic Acid.

^3^ partially-reduced Graphene Oxide.

^4^ SGU-29= copper silicate/ catalytic properties of the active sites provided by [CuO_4_] square planar units in SGU-29.

^5^ Tertio Butyl Hydro Peroxide.

^6^ HA= Halloysite Nanotube, HCL=Hydro Chloric Acid.

^7^ Ti-PILCs= acid-activated montmorillonite (PILC) and inter-spersed with titanium.

^8^ MoCT=Molybdate loaded Chitosan.

^9^ MoSi-500 =Molybdenum silicate spheres under non-aqueous conditions, calcined at 500 °C.

^10^ Cumene Hydroperoxide.

^11^ ligands supported on mesoporous silica and Cu(II) complex.

^12^ SBA.15. DAFO.Pd(II)^o^= 4, 5-diazofluorene-9-one (DAFO) to the mesoporous silica functionalized with aminopropyltriethoxysilane resulted in a material capable of absorbing Pd(II), SBA-15= Santa Barbara Amorphous-15.

^13^ Co(II) complexes with pentadendate Schiff-base ligand; N, N¢-bis(salicylidene)-2,6-pyridinediaminato, H_2_ [sal-2,6-py]; was entrapped in the nanocavity of Zeolite-Y by a two-step process in the liquid phase.

^14^ GO=graphene oxide.

^15^ TUD= three-dimensional sponge-like mesoporous materia.l

^16^ p(APTMACl)= Poly(3-acrylamidopropyl) trimethyl ammonium chloride.

^17^ nitrogen-doped carbons.

^18^ Anionic cobalt tetrasulfonatophenyl porphyrin (CoTPPS) intercalated NieAl LDH, LDH= layered double hydroxide.

^19^ PS=polymer‐anchored copper (II) complexes.

^20^ magnetic core–shell type Fe3O4@Chitosan-Schiff base Co(II).

^21^ MnPC= Manganese Porphyrin cage compound.

^22^ Gold nanoparticles were immobilizedthiolated chitosan derivative.

^23^ NPs=nano particles.

^24^ NR= nanorods.

^25^ CN= silver cyanide.

^26^ molybdenum and titanium species in the SBA-15 framework, SBA-15= Santa Barbara Amorphous-15.

^27^ Sulfonated polydivinylbenzene bamboo-like nanotube

^28^ PW=Venturello THA3 [PO4 {WO(O2)2}4], CNTs= carbon nanotubes

^29^ Dimethyl carbonate

^30^ Ti-containing Periodic Mesoporous Organosilica

^31^ Heteropolyacid-based poly (ionic liquid).

^32^ BPK= bipyridylketone

^33^ Fe-nano-catalyst has been covalently anchored on a modifie nanoscale SiO_2_/Al_2_O_3_.

**Table S3.** Commercial price and amount of chemical reagents used.

| Material | CAS. Number | Volume/Weight | Price ($/m^3^) |
| --- | --- | --- | --- |
| FeCl_2_·4H_2_O | 13478-10-9 | 0.3 g | 0.030 |
| FeCl_3_·6H_2_O | 10025-77-1 | 0.7 g | 0.110 |
| HCl | Dr Mojallali | 1 mL | 0.001 |
| NaOH | Arvandparak Company | 6 g | 0.053 |
| NH_4_ OH | Dr Mojallali | 4 mL | 0.013 |
| Ethanol | Simin Tak Company | 80 mL | 0.260 |
| TEOS | 78-10-4 | 0.6 mL | 0.050 |
| APTS | 13822-56-5 | 0.1 mL | 0.050 |
| Chitosan | 9012-76-4 | 0.1g | 0.017 |
| Acetic acid | 64-19-7 | 0.4 mL | 0.030 |
| Glutaraldehyde | 111-30-8 | 2 mL | 0.160 |
| Cobalt (II)-acetate tetrahydrate | 6147-53-1 | 0.5 | 0.029 |
|  |  | Total price ($g) | 0.823 |

**Table S4.** ANOVA for response surface quadratic model (analysis of variance).


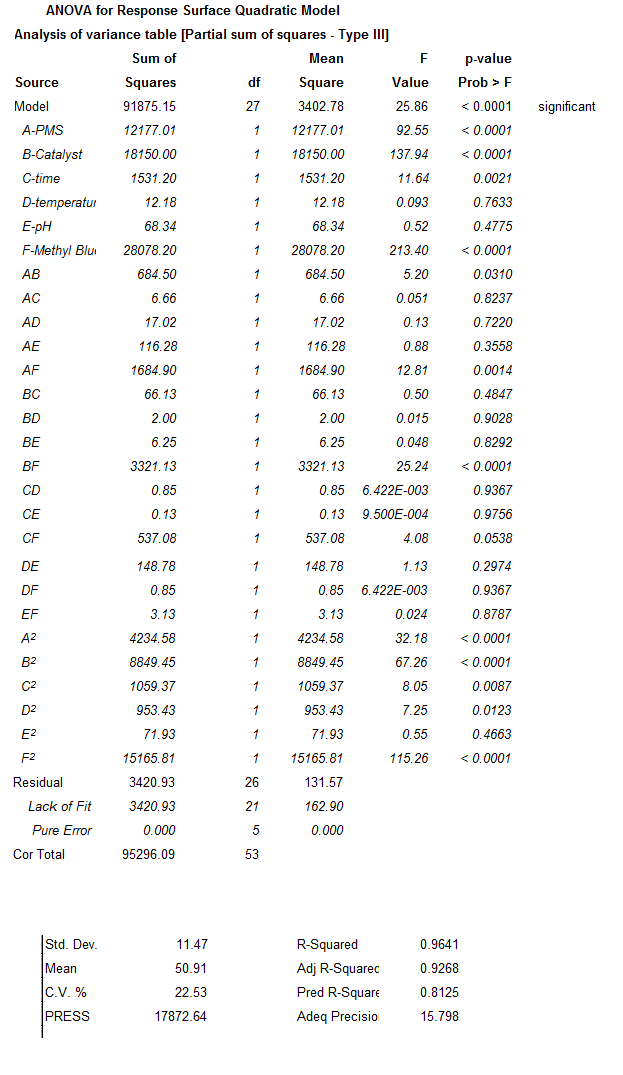


**
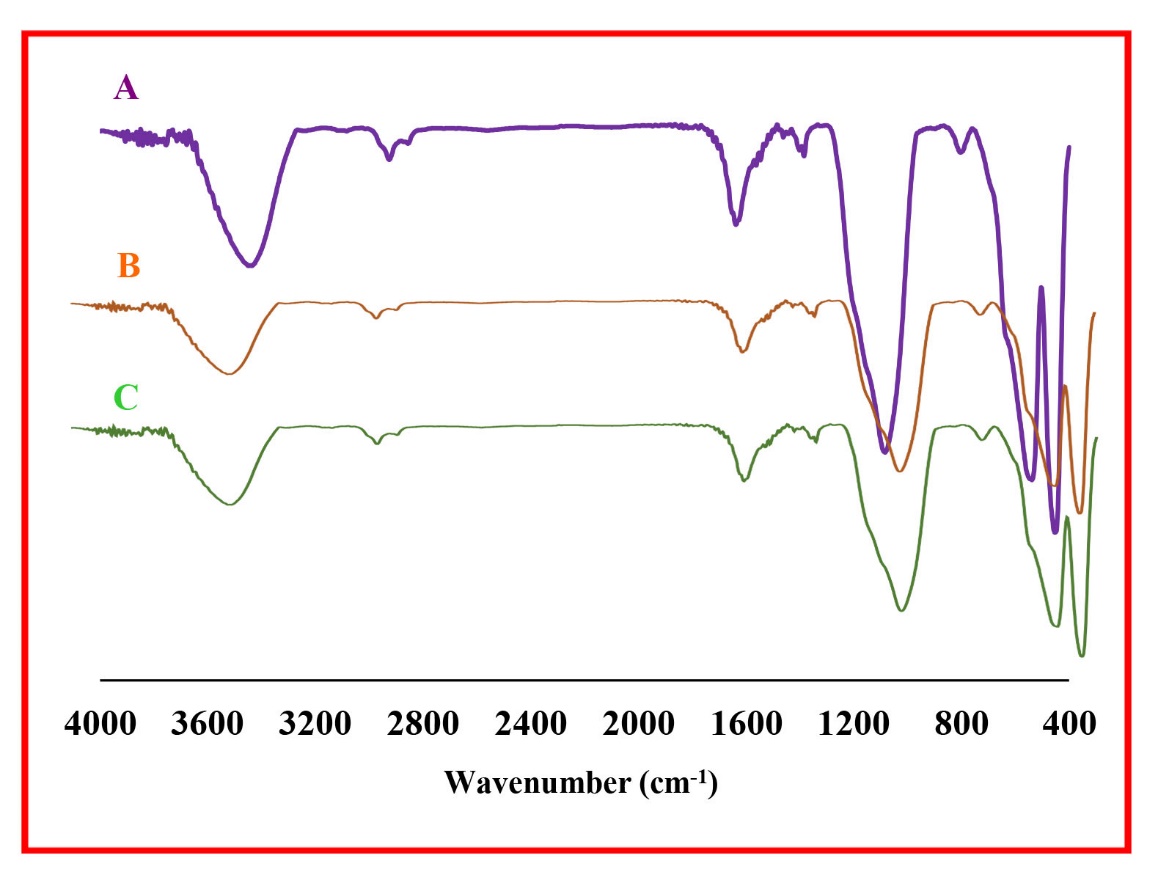
**

**Figure S1.** The FT-IR patterns of FS-(Am/g/Cs)@CoNP; fresh catalyst (A), after five cycles in MB degradation (B) and after six cycles in cyclohexene oxidation (C).

**
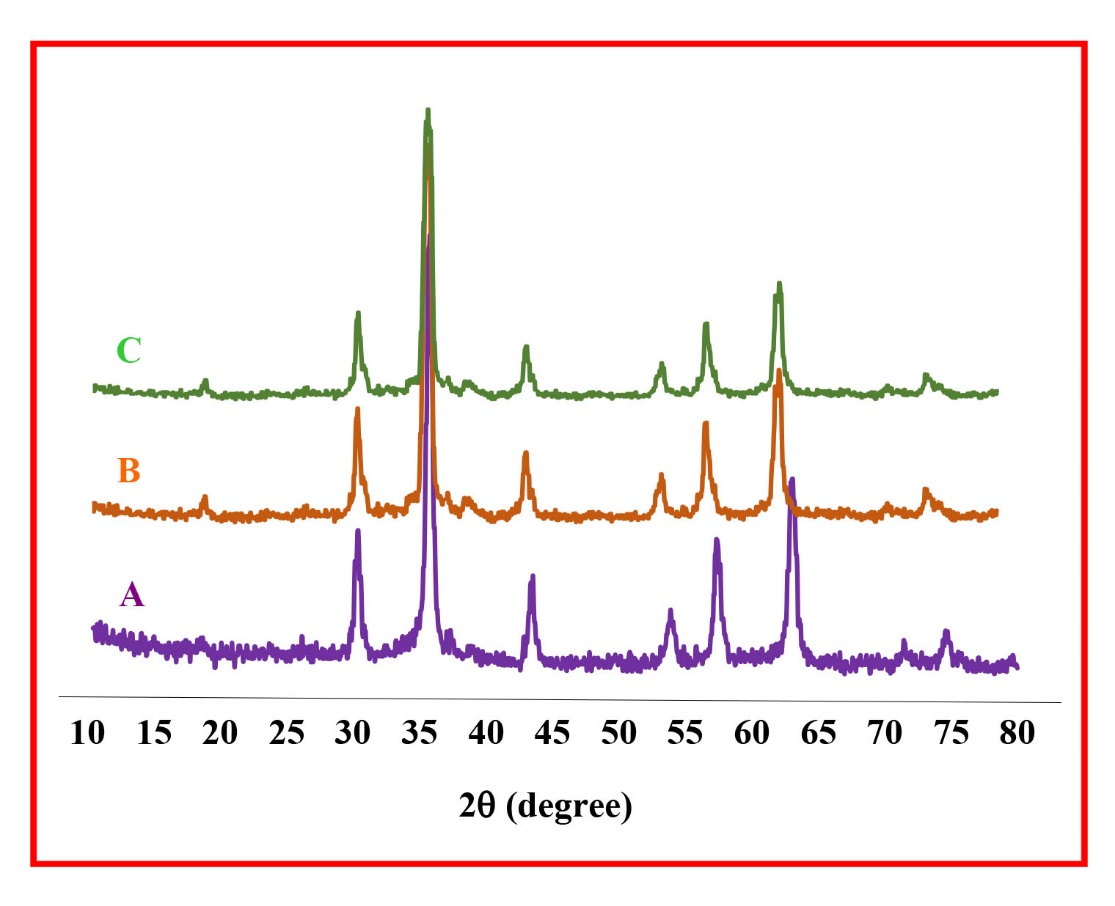
**

**Figure S2.** The XRD patterns of FS-(Am/g/Cs)@CoNP; fresh catalyst (A), after five cycles in MB degradation (B) and after six cycles in cyclohexene oxidation (C).

**References**

[1] Guo B, Ma J, Shi Y, Zheng K, Wu M, Ren G, Komarneni S. Co_3_O_4_/CoO ceramic catalyst: Bisulfite assisted catalytic degradation of methylene blue. Ceramics International. 2021 Oct 1;47(19):27617-23.

[2] Dong X, Lin Y, Ren G, Ma Y, Zhao L. Catalytic degradation of methylene blue by fenton-like oxidation of Ce-doped MOF. Colloids and Surfaces A: Physicochemical and Engineering Aspects. 2021 Jan 5; 608:125578.

[3] Lin S, Zhang T, Fu D, Zhou X. Utilization of magnesium resources in Salt Lake brine and catalytic degradation of dye wastewater by doping cobalt and nickel. Separation and Purification Technology. 2021 Sep 1; 270:118808.

[4] Wolski L, Sobańska K, Walkowiak A, Akhmetova K, Gryboś J, Frankowski M, Ziolek M, Pietrzyk P. Enhanced adsorption and degradation of methylene blue over mixed niobium-cerium oxide–Unraveling the synergy between Nb and Ce in advanced oxidation processes. Journal of Hazardous Materials. 2021 Aug 5; 415:125665.

[5] Zhang T, Ma Q, Zhou M, Li C, Sun J, Shi W, Ai S. Degradation of methylene blue by a heterogeneous Fenton reaction catalyzed by FeCo_2_O_4_-NC nanocomposites derived by ZIFs. Powder Technology. 2021 May 1; 383:212-9.

[6] Fayazi M. Preparation and characterization of carbon nanotubes/pyrite nanocomposite for degradation of methylene blue by a heterogeneous Fenton reaction. Journal of the Taiwan Institute of Chemical Engineers. 2021 Mar 1; 120:229-35.

[7] Jiao X, Li J, Wang C, Shi J, Jiao J, Zhang J, Lei Q, Xiao Z, Ye J, Guo S. Fenton-like reaction-induced degradation of Methylene Blue by using supermacroporous ferrimagnetic nanorings. InIOP Conference Series: Earth and Environmental Science 2021 Mar 1 (Vol. 687, No. 1, p. 012040). IOP Publishing.

[8] Dou R, Cheng H, Ma J, Qin Y, Kong Y, Komarneni S. Catalytic degradation of methylene blue through activation of bisulfite with CoO nanoparticles. Separation and Purification Technology. 2020 May 15; 239:116561.

[9] Ren G, Zhao K, Zhao L. A Fenton-like method using ZnO doped MIL-88A for degradation of methylene blue dyes. RSC Advances. 2020;10(66):39973-80.

[10] Peng Q, Tang X, Liu K, Luo X, He D, Dai Y, Huang G. High-efficiency catalysis of peroxymonosulfate by MgO for the degradation of organic pollutants. Minerals. 2019 Dec 18;10(1):2.

[11] Zhang K, Sun D, Ma C, Wang G, Dong X, Zhang X. Activation of peroxymonosulfate by CoFe_2_O_4_ loaded on metal-organic framework for the degradation of organic dye. Chemosphere. 2020 Feb 1; 241:125021.

[12] Dung NT, Thu TV, Van Nguyen T, Thuy BM, Hatsukano M, Higashimine K, Maenosono S, Zhong Z. Catalytic activation of peroxymonosulfate with manganese cobaltite nanoparticles for the degradation of organic dyes. RSC advances. 2020;10(7):3775-88.

[13] Huang F, Liu H, Wang H, Sun F, Chen T, Chen D, Xie Q, Zhou Y, Zhao Y. The activation of hematite for the catalytic hydrogen peroxide degradation of Methylene Blue. DESALINATION AND WATER TREATMENT. 2020 Oct 1; 201:383-92.

[14] Shen K, Cui Y, Zhang D, Liu M, Huang H, Sha X, Deng F, Zhou N, Zhang X, Wei Y. Biomimetic preparation of MoS_2_-Fe_3_O_4_ MNPs as heterogeneous catalysts for the degradation of methylene blue. Journal of Environmental Chemical Engineering. 2020 Oct 1;8(5):104125.

[15]. Junior OS, Monteiro AF, Oliveira JB, Araújo AM, Silva DG, Kulesza J, Barros BS. Coordination polymer-derived CuO catalysts for oxidative degradation of methylene blue. Materials Chemistry and Physics. 2019 Sep 1; 235:121737.

[16] Pan X, Cheng S, Su T, Zuo G, Zhao W, Qi X, Wei W, Dong W. Fenton-like catalyst Fe_3_O_4_@ polydopamine-MnO_2_ for enhancing removal of methylene blue in wastewater. Colloids and Surfaces B: Biointerfaces. 2019 Sep 1; 181:226-33.

[17] Li H, Xu S, Du J, Tang J, Zhou Q. Cu@ Co-MOFs as a novel catalyst of peroxymonosulfate for the efficient removal of methylene blue. RSC advances. 2019;9(17):9410-20.

[18] Zhao X, An QD, Xiao ZY, Zhai SR, Shi Z. One-step preparation of Fe_x_O_y_/N-GN/CNTs heterojunctions as a peroxymonosulfate activator for relatively highly-efficient methylene blue degradation. Chinese Journal of Catalysis. 2018 Nov 1;39(11):1842-53.

[19] Tao X, Wu Y, Wu Y, Zhang B, Sha H, Cha L, Liu N. Activated carbon‐supported cobalt molybdate as a heterogeneous catalyst to activate peroxymonosulfate for removal of organic dyes. Applied Organometallic Chemistry. 2018 Dec;32(12): e4572.

[20] Sajjadi S, Khataee A, Soltani RD, Bagheri N, Karimi A, Azar AE. Implementation of magnetic Fe_3_O_4_@ ZIF-8 nanocomposite to activate sodium percarbonate for highly effective degradation of organic compound in aqueous solution. Journal of Industrial and Engineering Chemistry. 2018 Dec 25; 68:406-15.

[21] Martínez F, Leo P, Orcajo G, Díaz-García M, Sanchez-Sanchez M, Calleja G. Sustainable Fe-BTC catalyst for efficient removal of mehylene blue by advanced fenton oxidation. Catalysis Today. 2018 Sep 1; 313:6-11.

[22] Wolski L, Ziolek M. Insight into pathways of methylene blue degradation with H_2_O_2_ over mono and bimetallic Nb, Zn oxides. Applied Catalysis B: Environmental. 2018 May 1; 224:634-47.

[23] Ye G, Zhou J, Huang R, Ke Y, Peng Y, Zhou Y, Weng Y, Ling C, Pan W. Magnetic sludge-based biochar derived from Fenton sludge as an efficient heterogeneous Fenton catalyst for degrading Methylene blue. Journal of Environmental Chemical Engineering. 2022 Apr 1;10(2):107242.

[24] Ezzayani K, Khelifa AB, Guesmi A, Hamadi NB, Abd El-Fattah W, Nasri H. Application of a new synthesized magnesium porphyrin complex in the degradation of methylene blue dye. Journal of Molecular Structure. 2022 Jun 15; 1258:132663.

[25] Guo H, Chen L, Zhang X, Chen H, Shao Y. Silicalite-1 Zeolite Encapsulated Fe Nanocatalyst for Fenton-like Degradation of Methylene Blue. Chinese Journal of Chemical Engineering. 2022 Mar 23.

[26] Wang L, Wang Y, Lv W, Yao Y. Activation of peroxymonosulfate by MgCoAl layered double hydroxide: Potential enhancement effects of catalyst morphology and coexisting anions. Chemosphere.

2022 Jan 1; 286:131640.

[27] Wu X, Sun D, Ma H, Ma C, Zhang X, Hao J. Activation of peroxymonosulfate by magnetic CuFe_2_O_4_@ ZIF-67 composite catalyst for the study on the degradation of methylene blue. Colloids and Surfaces A: Physicochemical and Engineering Aspects. 2022 Mar 20; 637:128278.

[28] Hu Y, Chen D, Wang S, Zhang R, Wang Y, Liu M. Activation of peroxymonosulfate by nitrogen-doped porous carbon for efficient degradation of organic pollutants in water: Performance and mechanism. Separation and Purification Technology. 2022 Jan 1; 280:119791.

[29] Luong TH, Nguyen TH, Nguyen BV, Nguyen NK, Nguyen TQ, Dang GH. Efficient degradation of methyl orange and methylene blue in aqueous solution using a novel Fenton-like catalyst of CuCo-ZIFs. Green Processing and Synthesis. 2022 Jan 1;11(1):71-83.

[30] Pandey NK, Li HB, Chudal L, Bui B, Amador E, Zhang MB, Yu HM, Chen ML, Luo X, Chen W. Exploration of copper-cysteamine nanoparticles as an efficient heterogeneous Fenton-like catalyst for wastewater treatment. Materials Today Physics. 2022 Jan 1; 22:100587.

[31] Li Z, Ning S, Zhu H, Wang X, Yin X, Fujita T, Wei Y. Novel NbCo-MOF as an advanced peroxymonosulfate catalyst for organic pollutants removal: Growth, performance and mechanism study. Chemosphere. 2022 Feb 1; 288:132600.

[32] El-Sawy AM, Gemeay AH, Helal AS, Salem MA. Catalytic degradation of methylene blue in aqueous solution by H_2_O_2_ and SiO_2_-NH_2_-Cu (II)@ SiO_2_ nanoparticles as catalyst. Journal of Molecular Liquids. 2021 Nov 1; 341:117422.

[33] Kabel KI, Mady AH, Rabie AM. Novel preparation of ferromanganese oxide based on hyperbranched polymer for peroxymonosulfate activation as a robust catalyst for the degradation of organic pollutants. Environmental Technology & Innovation. 2021 May 1; 22:101435

[34] Mia MS, Yao P, Zhu X, Lei X, Xing T, Chen G. Degradation of textile dyes from aqueous solution using tea-polyphenol/Fe loaded waste silk fabrics as Fenton-like catalysts. RSC advances. 2021;11(14):8290-305.

[35] Duan M, Wang X, Peng W, Liu D, Cheng Q, Yang Q. Co (II) Schiff Base Complex Supported on Nano‐Silica for the Aerobic Oxidation of Cyclohexene: Reaction Pathways and Overoxidation on the Experimental and Calculated Mechanism. Chemistry Select. 2021 Mar 26;6(12):2869-77.

[36] Rao BG, Sudarsanam P, Rao TV, Amin MH, Bhargava SK, Reddy BM. Highly dispersed MnO_x_ nanoparticles on shape-controlled SiO_2_ spheres for ecofriendly selective allylic oxidation of cyclohexene. Catalysis Letters. 2020 Oct;150(10):3023-35.

[37] Santibáñez L, Escalona N, Torres J, Kremer C, Cancino P, Spodine E. CuII-and CoII-Based MOFs: {[La_2_Cu_3_ (µ-H_2_O)(ODA) _6_ (H_2_O) _3_]∙ 3H_2_O} n and {[La_2_Co_3_ (ODA) _6_ (H_2_O) _6_]∙ 12H_2_O} n. The Relevance of Physicochemical Properties on the Catalytic Aerobic Oxidation of Cyclohexene. Catalysts. 2020 May;10(5):589.

[38] Pudukudy M, Jia Q, Dong Y, Yue Z, Shan S. Magnetically separable and reusable rGO/Fe _3_ O _4_ nanocomposites for the selective liquid phase oxidation of cyclohexene to 1, 2-cyclohexane diol. RSC advances. 2019;9(56):32517-34.

[39] Lee DH, Kim HS. Catalytic cyclohexene oxidation in the nano channels of a copper silicate material. Applied Catalysis A: General. 2019 Mar 25; 574:71-8.

[40] Azzi H, Rekkab-Hammoumraoui I, Chérif-Aouali L, Choukchou-Braham A. Mesoporous Co_3_O_4_ as a new catalyst for allylic oxidation of cyclohexene. Bulletin of Chemical Reaction Engineering & Catalysis. 2019;14(1):112.

[41] Li C, Zhao Y, Zhu T, Ruan J, Li G. Effective solvent-free oxidation of cyclohexene to allylic products with oxygen by mesoporous etched halloysite nanotube supported Co _2_+. RSC advances. 2018;8(27):14870-8.

[42] Dali A, Rekkab-Hammoumraoui I, El Korso S, Boudjema S, Choukchou-Braham A. Ruthenium-doped Titania-pillared Clay for The Selective Catalytic Oxidation of Cyclohexene: Influence of Ru Loading. Bulletin of Chemical Reaction Engineering & Catalysis. 2019 Sep 1;14(3):614-24.

[43] Advani JH, Bankar BD, Bajaj HC, Biradar AV. Chitosan supported molybdate nanoclusters as an efficient catalyst for oxidation of alkenes and alcohols. Cellulose. 2020 Oct;27(15):8769-83.

[44] Skoda D, Hanulikova B, Styskalik A, Vykoukal V, Machac P, Urbanek P, Bergerova ED, Simonikova L, Kuritka I. Non-aqueous synthesis of homogeneous molybdenum silicate microspheres and their application as heterogeneous catalysts in olefin epoxidation and selective aniline oxidation. Journal of Industrial and Engineering Chemistry. 2022 Mar 25; 107:320-32.

[45] Uruş S. Microwave assisted catalytic oxidation of cyclohexene, cyclohexane, cyclooctane and styrene with metal complexes of bis (azo-imine) ligands supported on mesoporous silica. Phosphorus, Sulfur, and Silicon and the Related Elements. 2022 Jan 20:1-1.

[46] Wu L, Han Y, Qi Y, Fu X, Chen R, Li J. Catalytic Oxidation of Cyclohexene by H_2_O_2_ Over Pd (II)-Complex Catalyst in a Heterogeneous System. Catalysis Letters. 2022 Jan 31:1-0.

[47] Jin M, Niu Q, Guo Z, Lv Z. Epoxidation of cyclohexene with H_2_O_2_ over efficient water‐tolerant heterogeneous catalysts composed of mono‐substituted phosphotungstic acid on co‐functionalized SBA‐15. Applied Organometallic Chemistry. 2019 Sep;33(9): e5115.

[48] Pour SR, Abdolmaleki A, Dinari M. Immobilization of new macrocyclic Schiff base copper complex on graphene oxide nanosheets and its catalytic activity for olefins epoxidation. Journal of Materials Science. 2019 Feb;54(4):2885-96.

[49] Hamdy MS, Alhanash AM, Benaissa M, Alsalme A, Alharthi FA, Al-Zaqri N. Rhodium Nanoparticles Incorporated Mesoporous Silica as an Active Catalyst for Cyclohexene Hydrogenation under Ambient Conditions. Catalysts. 2020 Aug;10(8):925.

[50] Ghorbanloo M, Moharramkhani N, Yazdely TM, Monfared HH. Cationic hydrogel and graphene oxide based cationic hydrogel with embedded palladium nanoparticles in the aerobic oxidation of olefins. Journal of Porous Materials. 2019 Apr;26(2):433-41.

[51] Denekamp IM, Antens M, Slot TK, Rothenberg G. Selective catalytic oxidation of cyclohexene with molecular oxygen: Radical versus nonradical pathways. ChemCatChem. 2018 Mar 7;10(5):1035-41.

[52] Radman R, Aouissi A, Al Kahtani A, Mekhamer W. Effect of CO_2_ on the oxidation of cyclohexene by H_2_O_2_ using Co1. 5PW12O40 catalyst. Petroleum Chemistry. 2017 Jan;57(1):79-84.

[53] Liu T, Cheng H, Lin W, Zhang C, Yu Y, Zhao F. Aerobic Catalytic Oxidation of Cyclohexene over TiZrCo Catalysts. Catalysts. 2016 Feb;6(2):24.

[54] Shen C, Ma J, Zhang T, Zhang S, Zhang C, Cheng H, Ge Y, Liu L, Tong Z, Zhang B. Intercalated cobalt porphyrin between layered double hydroxide nanosheets as an efficient and recyclable catalyst for aerobic epoxidation of alkenes. Applied Clay Science. 2020 Mar 15; 187:105478.

[55] Maurya A, Kesharwani N, Kachhap P, Mishra VK, Chaudhary N, Haldar C. Polymer‐anchored mononuclear and binuclear CuII Schiff‐base complexes: Impact of heterogenization on liquid phase catalytic oxidation of a series of alkenes. Applied Organometallic Chemistry. 2019 Sep;33(9): e5094.

[56] Cai X, Wang H, Zhang Q, Tong J, Lei Z. Magnetically recyclable core–shell Fe_3_O_4_@ Chitosan-Schiff base complexes as efficient catalysts for aerobic oxidation of cyclohexene under mild conditions. Journal of Molecular Catalysis A: Chemical. 2014 Mar 1; 383:217-24.

[57] Hakkoum A, Ameur N, Bachir R, Bedrane S, Choukchou-Braham A. Activity of Bimetallic Gold-Iron Catalysts in Adipic Acid Production by Direct Oxidation of Cyclohexene with Molecular Oxygen. Ann. Chim. -Sci. Matériaux. 2019 Oct; 43:299-304.

[58] Bernar I, Rutjes FP, Elemans JA, Nolte RJ. Aerobic epoxidation of low-molecular-weight and polymeric olefins by a supramolecular manganese porphyrin catalyst. Catalysts. 2019 Feb;9(2):195.

[59] Ghiaci M, Dorostkar N, Martínez-Huerta MV, Fierro JL, Moshiri P. Synthesis and characterization of gold nanoparticles supported on thiol functionalized chitosan for solvent-free oxidation of cyclohexene with molecular oxygen. Journal of Molecular Catalysis A: Chemical. 2013 Nov 15; 379:340-9.

[60] Qadir MI, Baptista DL, Dupont J. Effect of Support Nature on Ruthenium-Catalyzed Allylic Oxidation of Cycloalkenes. Catalysis Letters. 2022 Jan 6:1-8.

[61] Sudarsanam P, Singh N, Kalbande PN. Shape-controlled nanostructured MoO_3_/CeO_2_ catalysts for selective cyclohexene epoxidation. Catalysis Communications. 2022 Apr 1; 164:106433.

[62] Lu YJ, Janmanchi D, Natarajan T, Lin ZH, Wanna WH, Hsu IJ, Tzou DL, Ayalew Abay T, Yu SS. Silver Cyanide Powder‐Catalyzed Selective Epoxidation of Cyclohexene and Styrene with its Surface Activation by H_2_O_2_ (aq) and Assisted by CH_3_CN as a Non‐Innocent Solvent. ChemCatChem. 2022 May 2: e202200030.

[63] Zhang J, Zhang H, Liu L, Chen Z. The interaction of molybdenum and titanium in mesoporous materials for olefin epoxidation. Reaction Kinetics, Mechanisms and Catalysis. 2022 Jan 8:1-5.

[64] Quan X, Gu H, Hu C, Zhang Y, Li Y, Gao W, Li C. Sulfonated polydivinylbenzene bamboo-like nanotube stabilized Pickering emulsion for effective oxidation of olefins to 1, 2-diol. Journal of Colloid and Interface Science. 2022 Jan 15; 606:158-66.

[65] Evtushok VY, Lopatkin VA, Podyacheva OY, Kholdeeva OA. Immobilization of Polyoxometalates on Carbon Nanotubes: Tuning Catalyst Activity, Selectivity and Stability in H_2_O_2_-Based Oxidations. Catalysts. 2022 Apr 22;12(5):472.

[66] Awoke Y, Chebude Y, Díaz I. Ti-PMO materials as selective catalysts for the epoxidation of cyclohexene and vernonia oil. Catalysis Today. 2022 May 1; 390:246-57.

[67] Wu Y, Su M, Xiao Y, Guang B, Liu Y. Heteropolyacid-Based Poly (ionic liquid) s for the Selective Oxidation of Cyclohexene to 2-Cyclohexene-1-one. Industrial & Engineering Chemistry Research. 2021 Dec 29.

[68] Habibi D, Faraji AR, Arshadi M, Veisi H, Gil A. Manganese nanocatalyst and N-hydroxyphthalimide as an efficient catalytic system for selective oxidation of ethylbenzene, cyclohexene and oximes under aerobic condition. Journal of Molecular Catalysis A: Chemical. 2014 Feb 1; 382:41-54.

[69] Habibi D, Faraji AR, Arshadi M, Heydari S, Gil A. Efficient catalytic systems based on cobalt for oxidation of ethylbenzene, cyclohexene and oximes in the presence of N-hydroxyphthalimide. Applied Catalysis A: General. 2013 Sep 10; 466:282-92.

[70] Habibi D, Faraji AR, Arshadi M, Fierro JL. Characterization and catalytic activity of a novel Fe nano-catalyst as efficient heterogeneous catalyst for selective oxidation of ethylbenzene, cyclohexene, and benzylalcohol. Journal of Molecular Catalysis A: Chemical. 2013 Jun 1; 372:90-9.

[71] Amasha M, Baalbaki A, Ghauch A. A comparative study of the common persulfate activation techniques for the complete degradation of an NSAID: the case of ketoprofen. Chemical Engineering Journal. 2018 Oct 15; 350:395-410.

[72] L. Niu, G. Zhang, G. Xian, Z .Ren, T .Wei, Q. Li, Y. Zhang, Z. Zou. Tetracycline degradation by persulfate activated with magnetic γ-Fe_2_O_3_/CeO_2_ catalyst: performance, activation mechanism and degradation pathway. Separation and 259 (2021) 118156.
